# Antagonizing Retinoic Acid-Related-Orphan Receptor Gamma Activity Blocks the T Helper 17/Interleukin-17 Pathway Leading to Attenuated Pro-inflammatory Human Keratinocyte and Skin Responses

**DOI:** 10.3389/fimmu.2019.00577

**Published:** 2019-03-26

**Authors:** Florence Ecoeur, Jessica Weiss, Klemens Kaupmann, Samuel Hintermann, David Orain, Christine Guntermann

**Affiliations:** ^1^Autoimmunity, Transplantation, and Inflammation Disease Area, Novartis Institutes for BioMedical Research, Basel, Switzerland; ^2^Global Discovery Chemistry, Novartis Institutes for BioMedical Research, Basel, Switzerland

**Keywords:** retinoic acid receptor-related-orphan-receptor-gamma t, nuclear hormone receptor, T helper 17 differentiation, IL-17, autoimmunity, keratinocytes, skin inflammation

## Abstract

The nuclear hormone receptor retinoic acid receptor-related-orphan-receptor-gamma t (RORγt) is the key transcription factor required for Th17 cell differentiation and for production of IL-17 family cytokines by innate and adaptive immune cells. Dysregulated Th17 immune responses have been associated with the pathogenesis of several inflammatory and autoimmune diseases such as psoriasis, psoriatic arthritis, and ankylosing spondylitis. In this article, we describe the *in vitro* pharmacology of a potent and selective low molecular weight RORγt inhibitor identified after a structure-based hit-to-lead optimization effort. The compound interfered with co-activator binding to the RORγt ligand binding domain and impaired the transcriptional activity of RORγt as evidenced by blocked IL-17A secretion and RORE-mediated transactivation of a luciferase reporter gene. The inhibitor effectively reduced IL-17A production by human naive and memory T-cells and attenuated transcription of pro-inflammatory Th17 signature genes, such as *IL17F, IL22, IL26, IL23R*, and *CCR6*. The compound selectively suppressed the Th17/IL-17 pathway and did not interfere with polarization of other T helper cell lineages. Furthermore, the inhibitor was selective for RORγt and did not modify the transcriptional activity of the closely related family members RORα and RORβ. Using human keratinocytes cultured with supernatants from compound treated Th17 cells we showed that pharmacological inhibition of RORγt translated to suppressed IL-17-regulated gene expression in keratinocyte cell cultures. Furthermore, in *ex vivo* immersion skin cultures our RORγt inhibitor suppressed IL-17A production by Th17-skewed skin resident cells which correlated with reduced human β defensin 2 expression in the skin. Our data suggests that inhibiting RORγt transcriptional activity by a low molecular weight inhibitor may hold utility for the treatment of Th17/IL-17-mediated skin pathologies.

## Introduction

The Th17 lineage of T helper cells is essential for protective immunity against *Candida albicans* and against a variety of bacteria such as *Mycobacterium tuberculosis* and *Staphylococcus aureus* (1, 2). While critical in host immunity, Th17 cells which produce pro-inflammatory cytokines, mainly IL-17A, IL-17F, IL-22, and GM-CSF (3) have also been implicated in the pathogenesis of various autoimmune diseases including, psoriasis, psoriatic arthritis, ankylosing spondylitis, uveitis, and multiple sclerosis (4–7). There is mounting evidence that the Th17 pathway plays a central role in the pathophysiology of psoriasis. The Th17 signature cytokines IL-17A, IL-17F, and IL-22 can potentiate keratinocyte hyperproliferation and can activate keratinocytes to express various pro-inflammatory cytokines (IL-6, IL-8, TNF-α, IL-1β) and chemokines (CCL20, CCL20, CXCL1, CXCL2, CXCL3, CXCL5, and CXCL8). These mediators lead to enhanced recruitment of granulocytes and amplification of inflammation (8–10). Infiltration of Th17 cells and IL-17, IL-23, IL-22, and IL-23R expression levels are higher in psoriatic skin lesions compared to healthy control biopsies (11–14). The central importance of the Th17/IL-17 pathway in the pathogenesis of psoriasis and other inflammatory conditions has been confirmed by the impressive clinical efficacy following therapeutic intervention with antibodies neutralizing and blocking IL-17/IL-17 receptor interaction (7, 15–17).

RORγt and to a lesser extent RORα are required for the differentiation of Th17 cells and for promoting their pro-inflammatory function (18–21). RORγt controls the expression of the Th17 cytokines IL-17A, IL-17F, IL-22, IL-26 as well as IL-23 receptor and CCR6 (18, 22, 23). Expression of RORγt is not only confined to Th17 cells, but it also regulates cytokine production in other cell types, such as CD8^+^Tc17 cells, invariant natural killer T cells, ILC3 and γδ T-cells (24–28). All of these act in a coordinated fashion and contribute to autoimmune tissue inflammation (1, 25). RORγ deficient mice show diminished Th17/IL-17 *in vitro* responses and are protected in several animal models of autoimmune inflammatory diseases, such as experimental autoimmune encephalomyelitis, T-cell-transfer-mediated colitis and psoriasis-like skin inflammation (18, 29, 30). Pharmacological modulation of RORγt by low molecular weight inhibitors is therefore an attractive approach to inhibit the pro-inflammatory IL-17/IL-23 axis. Given that it is a nuclear hormone receptor, the activity of RORγt is regulated in a ligand-dependent manner. Numerous inhibitors targeting the ligand binding domain (LBD) of RORγt have been reported recently. These were effective in suppressing the IL-17 pathway and showed good efficacy in various inflammatory autoimmune disease models in rodents (31–33). Two isoforms of this nuclear receptor, RORγ and RORγt are known, which have identical LBDs. Because of their structural identities, compounds will inevitably bind to both of the RORγ/RORγt LBDs and consequently will inhibit the transcriptional activity of the two isoforms. In a previous communication, we published identification of a novel imidazopyridine series of potent and selective RORγt inhibitors by an extensive structure-based optimization campaign (34). Compound A [Cpd A; designated **34** in Hintermann et al. (34)] is a potent analog in this series that binds to the ligand binding pocket and inhibits RORγt by a typical “push-pull” mechanism by clashing with W317 if helix 12 is in the agonist position and by accepting a hydrogen bond from H479 (35). In the present study, we further characterized Cpd A focusing on various RORγt-dependent biochemical and cellular *in vitro* assays.

The inhibitor bound to the LBD of RORγt and impaired the interaction with a RIP140 co-activator peptide in a biochemical FRET assay. In a T-cell line that stably expressed RORγt, Cpd A repressed the RORγt transcriptional activity of multimerized ROR response elements (RORE)-driven luciferase gene without affecting RORγt recruitment to its cognate DNA RORE binding sites. Pharmacological inhibition of RORγt suppressed Th17 cell differentiation and RORγt target gene expression in primary human Th17 cells including *IL17A/F, IL22, IL26, IL23R*, and *CCR6*. The compound showed good selectivity against the IL-17 pathway and did not affect the differentiation of other T-helper cell subsets. Furthermore, Cpd A specifically targeted RORγt and did not interfere with the transcriptional activity of the related ROR family members, RORα or RORβ. The impact of RORγt inhibition on keratinocyte responses was analyzed by co-culturing the human HaCaT keratinocyte cell line with supernatants originating from compound treated Th17 cells. We identified IL-17 as the main Th17 cell derived cytokine that induced upregulation of the genes encoding NF-kappa-B inhibitor zeta (*NFKBIZ*) and psoriasis-associated mediators, including *CCL20, IL36G*, and the antimicrobial peptide human β Defensin 2 (*DEFB4A*) (36–41). RORγt inhibition during Th17 polarization diminished IL-17 production and incubation of keratinocytes with supernatants from compound treated Th17 cells resulted in attenuated expression of IL-17-induced genes in keratinocyte cultures.

Likewise, in human skin biopsies that were cultured with Th17-inducing cytokines, Cpd A down modulated IL-17A production by skin resident cells. Reduced IL-17A levels in compound treated skin samples correlated with impaired *DEFB4A* gene expression. These results provide strong evidence that pharmacological inhibition of RORγt by a low molecular weight antagonist may be effective in the treatment of IL-17A-mediated skin pathologies, such as psoriasis.

## Materials and Methods

### Human Study Approval

Blood from anonymized, healthy volunteers (20 ml per donor) was provided under informed consent and collected through the Novartis Tissue Donor Program (TRI0128) in accordance with the Swiss Human Research Act and approval of the responsible ethic committee (Ethikkommission Nordwest- und Zentralschweiz number: 329/13). Anonymized buffy coats from healthy volunteers were collected through the InterRegionale Blutspende of the Swiss Red Cross in Bern, Switzerland in compliance with article 2.2 lit. b of the Swiss Human Research Act.

Fresh skin discards obtained from healthy, female, Caucasian subjects undergoing aesthetic or reconstructive, abdominoplastic surgical resection were supplied by Alphenyx, Marseille, France. Studies were conducted in accordance with the ethical principles originating in the Declaration of Helsinki. Authorizations for the studies (AC-2014-2141 and IE-2014-749 for exportation) were obtained from the French Ministry of Research with the approval of the French Ethical Committee (Comité de Protection des Personnes). All subjects provided written informed consent before participating in the studies.

### Synthetic Chemistry

Cpd A, [(S)-N-(8-((4-(5-(tert-butyl)-1,2,4-oxadiazol-3-yl)-3-methylpiperazin-1-yl)methyl)-7-methylimidazo[1,2-a]pyridin-6-yl)-6-methylnicotinamide] was synthesized as described in Figure S1. Briefly, the 2-methylpyrimidine-5-carboxamide group of (S)-N-(8-((4-(5-(tert-butyl)-1,2,4-oxadiazol-3-yl)-3-methylpiperazin-1-yl)methyl)-7-methylimidazo[1,2-a]pyridin-6-yl)-2-methylpyrimidine-5-carboxamide [prepared as described in Hintermann et al. (34)] was cleaved with sodium hydroxide. The intermediate amine was subsequently converted into Cpd A by reaction with 6-methylnicotinic acid using T3P as the amide-coupling reagent.

The published RORγt inhibitor SR2211 (42) and the pan-JAK inhibitor CP690550 (43) were purchased from Tocris.

### Time Resolved Fluorescence Foerster Resonance Transfer Assay (TR-FRET)

Compound-mediated disruption of the interaction between the human RORγt-LBD (264-518, produced at Novartis) with a biotinylated RIP140 derived co-activator peptide (biotinyl-NH-Ahx-NSHQKVTLLQLLLGHKNEEN-CONH_2_, Thermo Scientific) was quantified by TR-FRET as previously described (34, 44). Briefly, Cpd A was incubated with a mixture consisting of 6 nM His_6_-tagged RORγt-LBD, 90 nM biotinylated RIP140 co-activator peptide, 0.45 nM Cy5-labeled streptavidin (GE Healthcare), 1.5 nM Europium-labeled anti-His_6_ antibody (Perkin Elmer). After 1 h of incubation, the emissions were measured at 615 and 665 nm. Concentration-response curves were generated and IC_50_ values were calculated using the GraphPad Prism software package.

### IL-17A Production by RORγt Transduced T-Cell Line

HUT78 T-cells stably expressing full-length eGFP-RORγt or eGFP-empty vector were stimulated and Cpd A-induced inhibition of IL-17A production was measured as described before (45). Briefly, HUT78 cells stably expressing RORγt (5 × 10^4^/well) were stimulated with PMA (8 ng/ml, Sigma) plus CD3 mAb (2.5 μg/ml, clone OKT-3, BioXcell) and incubated in X-vivo 15 medium containing 10% FCS with diluted Cpd A. After 48 h of incubation at 37°C, the supernatants were collected and IL-17A or IL-2 were quantified by ELISA according to the manufacturer‘s specifications (eBioscience or Biolegend).

### Generation of RORγt T-Cell Line Expressing Multimerized RORE Luciferase Reporter Gene

HUT78 T-cells stably expressing full-length eGFP-RORγt or eGFP-empty vector were generated as described before (45). Cells were transfected with plasmids containing 4 × ROREs inserted into the pGL4.17 luciferase Firefly reporter plasmid (kindly provided by Dr. Juergen Reinhardt, Novartis). As a control construct, a mutated or a non-functional form of 4 × RORE (Mut-RORE), was transfected in eGFP-RORγt HUT78 T-cells. The plasmids contained the following sequences where the bold nucleotides represent the putative RORγt binding site or the mutated form of the RORE:

Wild-type:

GGTAAGT**AGGTCA**TGGTAAGT**AGGTCA**TGGTAAGT**AGGTCA**TGGTAAGT**AGGTCA**TCGTGAC

CCATTCA**TCCAGT**ACCATTCA**TCCAGT**ACCATTCA**TCCAGT**ACCATTCA**TCCAGT**AGCACTG

Mutated:

GGTAAGT**ACCTCA**TGGTAAGT**ACCTCA**TGGTAAGT**ACCTCA**TGGTAAGT**ACCTCA**TCGTGAC

CCATTCA**TGGAGT**ACCATTCA**TGGGAGT**ACCATTCA**TGGAGT**ACCATTCA**TGGAGT**AGCACTG

Two days after transfection, cells were incubated in X-vivo 15 medium containing 10% FCS and 700 μg/ml Geneticin (Corning) and stable clones expressing the 4 × ROREs or mutated-ROREs were obtained by limiting dilution. The luciferase expression of the individual cell clones was measured using Luciferase detection buffer (Perkin Elmer) and the *RORC* mRNA expression of the selected clones were quantified by qRT-PCR.

Stable eGFP-RORγt HUT78 cells expressing either the 4 × ROREs or mutated-ROREs, eGFP-empty vector HUT78 cells expressing 4 × ROREs (5 × 10^4^/well) were stimulated with PMA (8 ng/ml, Sigma) plus CD3 mAb (2.5 μg/ml, clone OKT-3, Bioxell) and incubated in X-vivo 15 medium containing 10% FCS with diluted Cpd A. After 48 h of incubation at 37°C, the supernatants were removed and 40 μl of Luciferase detection buffer (Steady lite plus Reporter Gene assay system, Perkin Elmer) was added to the cells and further processed according to the manufacturer‘s instructions. The total luminescence signal of Firefly expression was measured by EnVision Multilabel Plate Reader (Perkin Elmer).

### RORα and RORβ GAL4 Reporter Gene Assays

Selectivity against other ROR family members was assessed in a GAL4 reporter gene assay as previously described (44). Briefly, Jurkat cells stably expressing the pGL4.35 plasmid (Promega) containing nine GAL4 binding sites inducing the transcription of the luciferase reporter gene were transiently transfected with the human RORα, RORβ or RORγt-LBD/GAL4-DBD constructs. After 24 h post-transfection, cells were incubated with Cpd A for an additional 24 h before luciferase activity was determined using an EnVision Multilabel Plate Reader (Perkin Elmer).

### Human CD4^+^ T-Cell Isolation and Differentiation

Total, naive and memory CD4^+^ T cells were isolated from Peripheral blood mononuclear cells (PBMCs) from healthy volunteers by density gradient centrifugation using Ficoll Paque (GE). Total, naive or memory CD4^+^ T-cells were prepared by immunomagnetic separation using the respective CD4^+^ T-cell enrichment kits resulting in untouched highly purified subsets (EasySep™, Stem Cell). Purity was assessed by flow cytometry and was between 95 and 98% for the total CD4^+^ T-cells. The purity of naive (CD45RA^+^CD45RO^−^) and memory (CD45RA^−^CD45RO^+^) CD4^+^T-cells ranged between 95 and 98%, respectively. For T-cell stimulation experiments, cells were treated as previously published (44). Briefly, T-cells were stimulated with plate-bound monoclonal antibodies against CD3 (1 μg/ml, clone OKT-3, BioXcell) and CD28 (1 μg/ml, clone CD28.2, BioLegend). For Th17 polarization assays, IL-6 (20 ng/ml), TGF-β1 (5 ng/ml), IL-1β (10 ng/ml), and IL-23 (10 ng/ml) were added. For Th1 polarization assays, IL-12 (10 ng/ml) and anti-IL-4 antibody (5 μg/ml), for Th2 polarizations, IL-4 (10 ng/ml) and anti-IFN-γ antibody (5 μg/ml) were added. For Th0 polarization assays, cells were only stimulated with CD3 and CD28 antibodies. Supernatants were collected after 3 or 7 days of incubation for total or naive/memory CD4^+^ T-cell polarizations, respectively. Cytokine concentrations were quantified by ELISA according to the manufacturer's instructions (eBioscience or BioLegend).

### FoxP3 Expression Analysis in Human CD4^+^ CD25 High T-Cells

PBMCs from freshly drawn heparinized blood originating from healthy volunteers were isolated by density gradient centrifugation using Ficoll Paque (GE). PBMCs (1 × 10^5^/well) were incubated for 96 h in the presence of DMSO or Cpd A (1 μM) together with anti-CD3 (3 ng/ml, clone OKT-3, BioXcell), anti-CD28 (30 ng/ml, clone CD28.2, BioLegend), TGF-β1 (0.2 ng/ml, Biolegend), IL-2 (15 ng/ml, Biolegend), anti-IFN-γ antibody (5 μg/ml, Biolegend), and anti-IL-4 (5 μg/ml, Biolegend). Cells were prepared for CD4/CD25 surface staining, followed by intracellular FoxP3 staining.

### Surface and Intracellular Staining for Flow Cytometry

Th17 cells obtained from *in vitro* cultures after 72 h were incubated for 4 h at 37°C with 5 ng/ml of PMA (Sigma) and 500 ng/ml of ionomycin (Calbiochem) in the presence of 10 μg/ml Brefeldin A (Sigma). The cells were washed, fixed and permeabilized in Cytofix/Cytoperm buffer (BD Biosciences) according to the manufacturer‘s instructions prior to intracellular staining with PE labeled IL-17A antibody (eBioscience) and APC-labeled IFN-γ antibody (eBioscience).

For FoxP3 detection within the CD4^+^ CD25 high T-cells, surface staining was performed for 20 min with FITC labeled CD4 antibody and anti-CD25 BV421 antibody (BD Bioscience). After surface staining, the cells were washed, fixed and permeabilized according to the manufacturer's instructions (eBioscience) prior to intracellular staining with APC labeled FoxP3 antibody (eBioscience). Data were acquired on a LSR Fortessa (BD Bioscience) and analyzed using FlowJo software (Tree Star). FoxP3 expression was determined in gated CD4^+^ CD25 high T-cells that were treated with DMSO or with Cpd A.

### Analysis of Phospho-STAT3 Activation by Flow Cytometry

To assess whether Cpd A affected STAT3 activity we induced STAT3 phosphorylation in Ba/F3 cells stably expressing the human IL-21 receptor (kindly provided by Dr. Max Warncke, Novartis). Cells were pre-incubated for 1 h with 10 μM of Cpd A or with 1 μM of the pan-JAK inhibitor CP690550. To induce STAT3 activity, the cells were stimulated for 1.5 h with recombinant human IL-21 (3 nM), followed by two washing steps using buffer containing PBS/0.5% BSA. The cells were fixed with 2% paraformaldehyde for 15 min at room temperature, washed and resuspended in ice-cold 90% methanol solution and stored overnight at −20°C. The samples were washed and stained for 1 h with an Alexa 647-labeled anti-phospho (pY705)-STAT3 antibody (BD biosciences). After two washes the cells were analyzed using a LSR Fortessa (BD Bioscience) and analyzed using FlowJo software (Tree Star).

### RORE Oligonucleotide Pull-Down Assays

In order to detect RORγt binding to its cognate DNA binding sites, biotinylated oligonucleotide probes containing putative 4 × RORE or mutated-RORE DNA sequences were synthesized (Microsynth, Switzerland) and annealed in order to form a double stranded DNA probe. The probes contained the following sequences (where the bold nucleotides represent a putative RORγt binding site or the mutated form of the RORE):

RORE sense strand: 5'GGTAAGT**AGGTCA**TGGTAAGT**AGGTCA**TGGTAAGT**AGGTCA**TGGTAAGT**AGGTCA**TCGTGAC-3**'** Biotin

RORE antisense strand: 3'-CCATTCA**TCCAGT**ACCATTCA**TCCAGT**ACCATTCA**TCCAGT**ACCATTCA**TCCAGT**AGCACTG-5**'**

RORE mutated sense strand: **5'**-GGTAAGT**ACCTCA**TGGTAAGT**ACCTCA**TGGTAAGT**ACCTCA**TGGTAAGT**ACCTCA**TCGTGAC-3' Biotin

RORE mutated antisense strand: 3'-CCATTCA**TGGAGT**ACCATTCA**TGGGAGT**ACCATTCA**TGGAGT**ACCATTCA**TGGAGT**AGCACTG-5'

Nuclear extracts originating from HUT78 cells stably expressing eGFP-RORγt were prepared as previously described (45). Briefly, cells were incubated with Cpd A (10 μM final concentration) for 0.5 h followed by a 2 h-stimulation period using CD3 antibody (2.5 μg/ml, clone OKT-3, BioXcell) plus PMA (8 ng/ml, Sigma). Nuclear lysates were generated using a nuclear extraction kit (Pierce, Thermo scientific), followed by a pre-clearing step with streptavidin-sepharose beads (GE healthcare Amersham) for 1 h at 4°C. The supernatants were incubated with biotinylated double-stranded RORE or with mutated RORE probes (10 nM final concentration) for 1 h at 4°C, followed by addition of a 50% slurry of streptavidin-sepharose beads for 1 h. The bound complexes were washed five times with 1 ml buffer consisting of 50 mM Tris-HCl, 1 mM EDTA, 1% NP-40, 150 mM NaCl and protease inhibitors (Roche). After the final wash, the pull-downs were resuspended in 2 × SDS-PAGE sample buffer (Sigma), boiled and used for SDS-PAGE and Western blotting. The samples were immunoblotted with a polyclonal RORγ-specific antibody (Novus Biologicals). As a loading control, nuclear extracts containing 2 × 10^6^ cell equivalent were blotted with RORγ-specific antibody and with GAPDH-specific antibody (clone 14C10, Cell Signaling). Protein bands were visualized by enhanced chemiluminescence (Western bright Quantum, Advansta) followed by detection of the band using a Western blot imaging system (Fusion FX, Vilber Lourmat).

### Analysis of IL-17-Driven Keratinocyte Responses

The HaCaT keratinocyte cell line was purchased from AddexBio and was maintained in RPMI 1640 medium supplemented with Glutamax, Penicillin/Streptomycin (all from Gibco) and 10% FCS (PAA). Normal primary human epidermal keratinocytes (NHEKs) from a male and female caucasian adult were purchased from Lonza and PromoCell, respectively. Cells were cultured in Keratinocyte basal growth medium (KBM; Lonza) containing KGM2 growth supplements including insulin, human epidermal growth factor, bovine pituitary extract, hydrocortisone, epinephrine, transferrin, and gentamicin/amphotericin B (Lonza). HaCaT cells were incubated in a 96-well-microtiter plate (2 × 10^4^ cells/well) with Th17 cytokine-containing supernatants obtained from total CD4^+^ T-cells that were polarized for 72 h in the presence of anti-CD3/CD28 antibodies and a Th17 polarization cocktail as described above. The conditioned supernatants were generated in the presence of a pharmacologically active Cpd A concentration (10 μM) and the keratinocyte responses were compared with an inactive Cpd A concentration (0.13 nM). Alternatively, HaCaT and NHEKs (1 × 10^4^ cells/well) were used in spike-in experiments using recombinant human cytokines at the concentrations determined in the Th17 supernatants exposed to bioactive concentration (10 μM) and inactive Cpd A concentration (0.13 nM). Cytokine concentrations in supernatants were determined from representative experiments and they contained approximately 150 pg/ml or 600 pg/ml of IL-17 in 10 μM or in 0.13 nM treated samples, respectively. The TNF-α concentration was similar in supernatants exposed to low or high Cpd A concentrations and was 4 ng/ml. To study the impact of the IL-17 cytokine, IL-17 in the supernatants and in the spike-in cocktails was neutralized by a 0.5 h pre-incubation with an IL-17-specific antibody [0.75 μg/ml; Secukinumab (Cosentyx®, Novartis)]. All cultures were incubated in triplicates and after a 24 h incubation period, HaCaT cells (6 × 10^4^ cells/sample) and NHEKs (1 × 10^5^ cells/sample) were harvested and further processed for qRT-PCR analysis.

### Human Skin Biopsies and *ex-vivo* Stimulation of Skin-Resident Immunocompetent Cells

Full-thickness skin punch biopsies (8 mm diameter) were taken from surgical discards. Briefly, subcutaneous fat was removed from the skin ranging in size between 150 and 200 cm^2^ and 8 mm punches were obtained. Samples were incubated in IMDM medium supplemented with Glutamax, 10% FCS, 1% non-essential amino acids, 1 mM Sodium pyruvate, Penicillin/Streptomycin and Amphotericin B (2.5 μg/ml). Incubations were done in the presence of various concentrations of Cpd A or DMSO vehicle control together with a Th17-inducing cocktail consisting of IL-6 (20 ng/ml), TGF-β1 (5 ng/ml), IL-1β (10 ng/ml), IL-23 (10 ng/ml), anti-IL-4/IFNγ (5 μg/ml), and anti-CD3/CD28 antibodies (1 μg/ml). On day 4, supernatants were harvested and analyzed for IL-17 cytokine expression, whereas skin punch biopsies were snap frozen, stored at −80°C and further processed for qRT-PCR analysis.

### Quantitative RT-PCR

Total RNA from human Th17 cells (6 × 10^5^ cells), RORγt-RORE HUT78 cells (4 × 10^5^ cells) and HaCaT cells (6 × 10^4^ cells) was extracted using RNeasy kit including a DNAse I digestion step according to the manufacturer‘s protocol (Qiagen). Frozen skin biopsies (8 mm punches) were resuspended in RLT buffer (Qiagen) containing 1% β-mercaptoethanol and homogenized using a Precellys-Cryolys device (Bertin Instruments) according to the manufacturer's instructions. cDNAs were prepared with the High Capacity cDNA Reverse Transcription Kit (Applied Biosystems). Quantitative RT-PCR analysis was performed using a TaqManViiA7 (Applied Biosystems). All runs were accompanied by the internal control gene human β-glucoronidase (*Gus*) (431088E). The samples were run in triplicate or in hexaplicate and normalized to *Gus* using the ΔΔCt method to provide arbitrary units representing relative expression levels. The following Taqman probes were used for RT-PCR (Thermo Scientific): *RORC* (Hs01076112_m1), *IL17A* (Hs00936345_m1), *IL22* (Hs01574154_m1), *IL26* (Hs00218189_m1), *IL23R* (Hs00332759_m1), *CCR6* (Hs00218189_m1), *NFKBIZ* (Hs00355476_m), *DEFB4A* (Hs00823638_m1), *IL36G* (Hs00219742_m1), and *CCL20* (Hs00355476_m1).

### Statistics

The means were compared using both ANOVA followed by Dunnett's test for multiple comparisons (GraphPad Software Corporation Inc. CA). A *p* < 0.05 was considered statistically significant.

## Results

### Identification of a Potent RORγt Inhibitor

**Figure 1:**
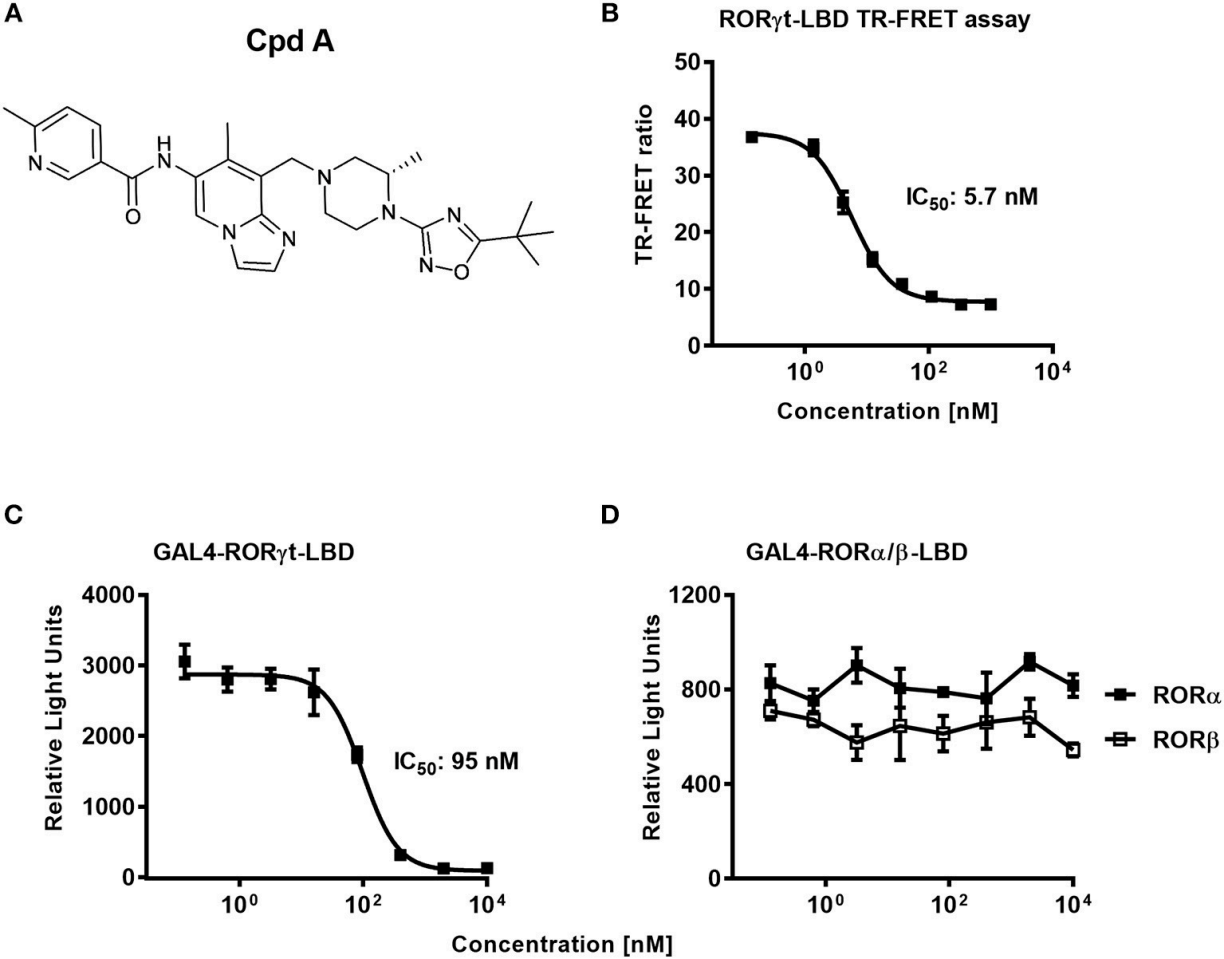
The RORγt inhibitor Cpd A prevents co-activator peptide binding to the ligand binding domain of RORγt and inhibits transcriptional activity of RORγt in a GAL4 reporter gene assay in Jurkat T-cells. **(A)** Chemical structure of the low molecular weight RORγt inhibitor Cpd A. **(B)** Biochemical TR-FRET assay quantifying the interaction between a RIP140 co-factor peptide with the human RORγt-LBD. Representative concentration-dependent curve from at least five independent experiments with duplicate readings is shown. The mean IC_50_ value ± SD of the published RORγt inhibitor SR2211 was 4.3 ± 4.3 nM as determined in two experiments. **(C,D)** Cpd A is a potent and selective RORγt inhibitor in a cellular RORγt-LBD GAL4 transactivation assay and has no impact on the transcriptional activity of the closely related RORα and RORβ nuclear hormone receptors. All experiments were performed three times with similar results. Error bars represent the SD. The mean IC_50_ value obtained for SR2211 in the RORγt-LBD GAL4 transactivation assay was 418 ± 84 nM (*n* = 2).

In a previous communication, we reported the discovery of a series of imidazopyridine RORγt inhibitors by a structure-based hit-to-lead optimization effort (34). Cpd A, which is an advanced representative of this series (Figure 1A), was chosen for investigating further *in vitro* RORγt-related pharmacology. Nuclear hormone receptors interact with co-activators and co-repressors to control gene transcription (46). We therefore examined in a biochemical TR-FRET assay, the effect of Cpd A to interfere with the RIP140 co-activator peptide binding to the human RORγt-LBD. Consistent with our previous studies, Cpd A disrupted the interaction of the RIP140 peptide with the RORγt-LBD in a concentration-dependent manner with an IC_50_ value of 5.7 nM. Similar IC_50_ values in the RIP140 co-activator displacement assay were obtained when the published RORγt inhibitor SR2211 was used (Figure 1B).

**Figure 2:**
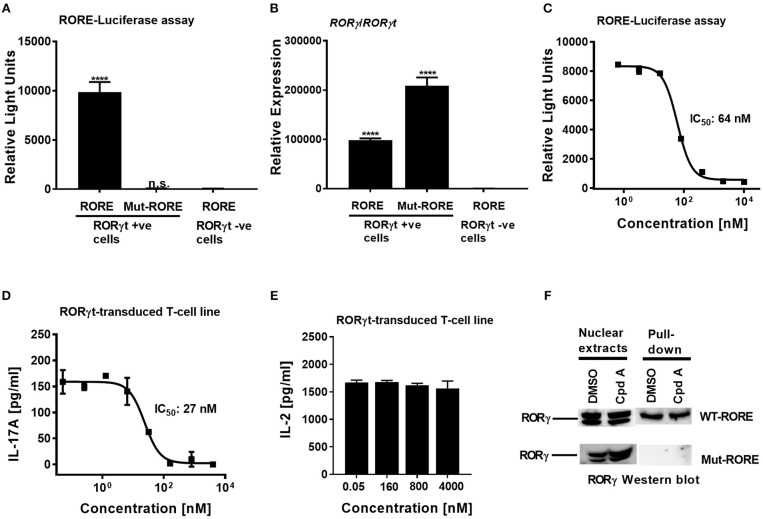
Cpd A potently inhibits transcriptional activity of human RORγt in the full-length context in T-cells. **(A)** Full-length RORγt drives wild-type RORE-dependent activation of a reporter gene in HUT78 T-cells expressing RORγt, but not in RORγt negative cells. HUT78 cells stably expressing RORγt or empty control vector were transfected with 4 x RORE or mutated RORE-luciferase reporter constructs, followed by stimulation with CD3 antibody/PMA for 48 h and by quantifying luciferase activity. **(B)** Gene expression level of *ROR*γ/*ROR*γ*t* in HUT78 T-cells stably expressing RORγt or control vector together with RORE or mutated RORE-reporter genes. **(C)** Cpd A potently blocked RORE-mediated transcription of the luciferase reporter gene. HUT78 T-cells were stimulated with CD3 antibody plus PMA for 48 h followed by measurement of luciferase activity. **(D)** HUT78 T-cells stably expressing RORγt were incubated with serial dilutions of Cpd A at the beginning of the stimulation with PMA and anti-CD3 antibody and after 48 h IL-17A or IL-2 **(E)** concentrations were measured by ELISA. Representative examples of concentration-response curves from three independent experiments are shown. The average IC_50_ value obtained for SR2211 in the RORγt-IL-17A inhibition assay was 257 ± 181 nM (*n* = 2). **(F)** RORγt transduced HUT78 T-cells were pre-incubated with Cpd A (10 μM) or DMSO, followed by a 2 h stimulation with anti-CD3 antibody/PMA and nuclear extracts were prepared. Nuclear extracts were subjected to pull-down experiments using mutated (Mut-ROREs) or wild-type (WT ROREs) biotinylated RORE oligonucleotides followed by immobilization of complexes with streptavidin Sepharose beads. Nuclear extracts (left panel) and RORE pull-down complexes (right panel) were subjected to SDS PAGE followed by RORγ Western blot analysis. Results shown are representative of three experiments with triplicate readings with similar results, except for **(B)** which originates from a single experiment. Error bars show the SD. Statistical analyses were performed using one way ANOVA Dunnett's test, ****p < 0.0001.

To confirm that Cpd A represses the transcriptional activity of RORγt in a cellular context, we performed a RORγt-LBD/GAL4 based transactivation assay in Jurkat T-cells where luciferase reporter gene transcription is driven by the GAL4-RORγt-LBD fusion construct. We could demonstrate that Cpd A possessed good activity in a T-cell environment resulting in complete inhibition of GAL4 promoter activity with an IC_50_ value of 95 nM, while the SR2211 compound was less potent (Figure 1C). To address the potency of Cpd A against the closely related ROR family members RORα and RORβ we performed LBD-GAL4 reporter gene assays and could show that the inhibitor lacked activity toward the RORα and RORβ isoforms, indicating that the compound acted selectively against RORγt (Figure 1D). To confirm that Cpd A inhibits full-length RORγt, we expressed the full-length version of RORγt along with a 4 × RORE response element that induces luciferase gene expression once the elements are occupied by RORγt. For these assays, we used HUT78 T-cells that stably expressed human RORγt, as previously described (45), which were transfected with plasmids containing the consensus 4 × RORE DNA binding elements (AGGTCA) driving the luciferase reporter gene activity. As a control, HUT78-RORγt expressing cells were stably transfected with non-functional, mutated forms of the RORE (ACCTCA). HUT78 cells that did not express RORγt that contained the 4 × RORE did not have any detectable luciferase activity, whereas in RORγt expressing cells RORE-driven reporter gene activity was significantly up-regulated (Figure 2A). Similarly, RORγt expressing cells transfected with the mutated version of the ROREs failed to show any luciferase reporter activity (Figure 2A). Expression of RORγt mRNA was not altered in cells transfected with the functional or mutated RORE versions ruling out that lack of transcriptional activity in the clone carrying the mutated ROREs occurs as a result of reduced RORγt expression (Figure 2B). Treatment with Cpd A significantly repressed 4 × RORE-driven luciferase activity with an IC_50_ value of 64 nM (Figure 2C). To further examine the activity of the inhibitor in a more natural setting, we determined whether Cpd A could inhibit endogenous IL-17A secretion in the HUT78 T-cell line that stably expressed full-length RORγt. When these cells are stimulated with anti-CD3 Ab plus PMA, IL-17A secretion is dependent on RORγt expression (45). Pharmacological inhibition of RORγt by Cpd A resulted in complete attenuation of IL-17A production in a concentration-dependent manner with an IC_50_ value of 27 nM, whereas the IC_50_ value for SR2211 was ~10-fold higher (Figure 2D). The observed inhibition could be due to non-specific suppression of CD3/PMA-induced T-cell signaling. However, IL-2 cytokine production which is not controlled by RORγt was not affected by Cpd A (Figure 2E) indicating that the inhibitor interfered exclusively with the IL-17 pathway.

In summary, Cpd A showed potent and selective inhibition of RORγt-mediated reporter gene transcription and of IL-17A production in various T-cell assays expressing either full-length RORγt or the LBD of RORγt. By contrast, SR2211 was in all our cellular assays less potent than Cpd A.

### Cpd A Does Not Perturb RORE DNA Occupancy by RORγt

In order to gain mechanistic insight of how Cpd A inhibits RORγt transcriptional activity it was further investigated whether the compound interfered with the ability of RORγt to bind to its cognate RORE DNA binding sites. Based on the results in the RORE-dependent transactivation assay described above (Figures 2A–C), the compound may inhibit the transcriptional activity of RORγt by disrupting binding to the ROREs located at the target DNA regulatory sites or by interfering with co-factor assembly crucial for target gene transcription without affecting binding to the ROREs.

We explored whether Cpd A may adversely affect the DNA-binding activity of RORγt by assessing the interaction between this nuclear hormone receptor and the ROR elements. In a previous communication, we reported that recruitment of RORγt to the ROREs occurs after stimulation of RORγt HUT78 T-cells with anti-CD3/PMA (45). Using this cellular model, nuclear extracts were prepared from stimulated RORγt HUT78 T-cell transfectants and pull-down studies were conducted using the DNA oligonucleotides containing the RORE sequences (AGGTCA) or the non-functional RORE sequences (ACCTCA). Western blot analysis of the pull-down experiments revealed that RORγt occupied only the functional RORE sequences, whereas RORγt binding was not detectable when the mutated versions of the RORE oligonucleotides were used as a specificity control (Figure 2F). RORγt association with the ROR elements was still intact in cells that were treated with the inhibitor and was similar compared to DMSO treated samples (Figure 2F). These results indicate that Cpd A inhibits the activity of RORγt through interference with co-factor assembly and/or other transcription factor interactions rather than interfering with the DNA-binding capacity of RORγt.

### Selective Inhibition of Human Th17 Cell Differentiation by Cpd A

**Figure 3:**
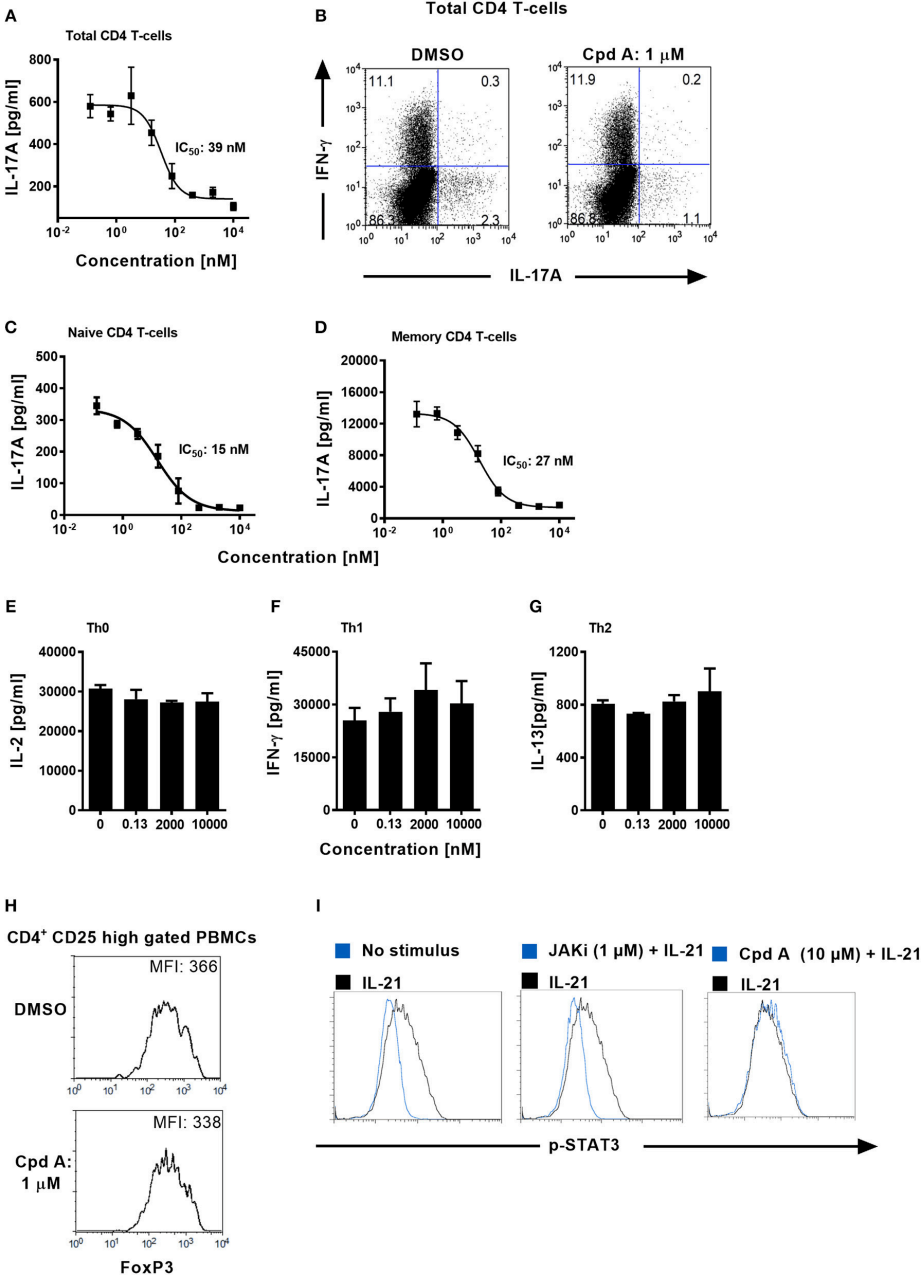
Cpd A specifically blocks human Th17 cell polarization, while leaving Th0, Th1, or Th2 T-cell polarization, STAT3 phosphorylation and FoxP3 expression intact. **(A)** Total human CD4^+^ T-cells were isolated from buffy coats and were polarized toward the Th17 phenotype for 72 h in the presence of the RORγt inhibitor and IL-17A production was quantified by ELISA. The mean IC_50_ value ± SD of the published RORγt inhibitor SR2211 from two experiments assay was 1475 ± 739 nM (*n* = 2). **(B)** Total CD4^+^ T-cells were polarized toward the Th17 phenotype for 72 h, incubated with PMA/Ionomycin and Brefeldin A for 4 h and frequencies of IL-17A/IFN-γ-producing CD4^+^ T-cells were determined by intracellular FACS. Dot plots of IL-17A/IFN-γ producing cells of gated CD4^+^ T-cells are shown. **(C,D)** Naive and memory human CD4^+^ T-cells were isolated and were polarized toward the Th17 phenotype for 72 h in the presence of the RORγt inhibitor and IL-17A production was quantified **(E–G)** Total CD4^+^ T-cells were stimulated with anti-CD3 plus anti-CD28 antibodies only (Th0), incubated with IL-12 and anti-IL-4 antibody (Th1) or with IL-4 and anti-IFN-γ antibody (Th2). After 48 h, Th subset signature cytokines including IL-2, IFN-γ, and IL-13 were analyzed by ELISAs. All results are representative from three experiments containing three technical replicates. Error bars show the SD. **(H)** PBMCs were stimulated for 96 h with CD3+CD28-specific antibodies together with IL-2, TGF-β, and IL-4 + IFN-γ-specific antibodies and FoxP3 expression was analyzed by intracellular flow cytometry. Histoplots of FoxP3 expression profiles within gated CD4^+^ CD25 high T-cells are shown. All results are representative from three experiments. **(I)** Ba/F3 cells stably transfected with the human IL-21 receptor were pre-incubated for 1 h either with DMSO, Cpd A or with a JAK inhibitor followed by stimulation with human IL-21. The samples were fixed followed by intracellular staining using an anti-phospho (pY705)-STAT3 antibody. Histoplot overlays showing p-STAT3 expression in non-stimulated vs. IL-21-treated or compound-treated cells are shown. Results are representative from three experiments.

We next addressed whether Cpd A impacted Th17 differentiation in primary human T-cells. Total CD4^+^ T-cells originating from healthy volunteers were isolated and stimulated with anti-CD3 plus anti-CD28 specific antibodies together with a Th17 skewing cytokine cocktail in the presence of Cpd A. The inhibitor showed better potency than SR2211 and potently suppressed IL-17A secretion in a concentration-dependent manner with an IC_50_ value of 39 nM (Figure 3A). Likewise, intracellular staining followed by flow cytometric analysis revealed that Cpd A reduced the frequencies of IL-17A^+^ cells within the CD4^+^ T-cell population during the 3-day Th17 differentiation culture period while the frequencies of INF-γ^+^ cells were not affected (Figure 3B). When naive or memory human CD4^+^ T-cells were differentiated for 7 days toward the Th17 cell phenotype, Cpd A was potent and impaired IL-17A cytokine secretion in both CD4^+^ T-cell subsets with IC_50_ values of 15 and 27 nM for naive and memory CD4^+^ T-cells, respectively (Figures 3C,D). While Cpd A impaired IL-17A secretion in CD4^+^ T-cells that were skewed toward the Th17 phenotype, it did not have any impact on Th0, Th1, or Th2 signature cytokine production (IL-2, IFN-γ, and IL-13, respectively), indicating that the compound inhibited selectively the Th17 pathway (Figures 3E–G).

Regulatory T-cells (Tregs) and Th17 cells are reciprocally regulated, depending on the balance between the expression of RORγt and FoxP3 and on the cytokine environment (47, 48). It is possible that RORγt inhibition mediated by Cpd A may skew the Th17/Treg cell ratio toward the Treg pathway. We determined whether our RORγt inhibitor had an impact on the expression of the transcriptional regulator FoxP3 which is crucial for the development and inhibitory function of regulatory T-cells (Tregs) (49). PBMCs were cultured for 96 h under Treg inducing conditions in the presence of Cpd A and FoxP3 expression among CD4^+^CD25high T-cells was monitored. Flow cytometric analysis of gated CD4^+^CD25high T-cells revealed high expression of FoxP3 in these cells and incubation of our RORγt inhibitor did not result in altered FoxP3 expression as shown by similar mean fluorescence intensities between DMSO or inhibitor treated cells (Figure 3H), indicating that Cpd A does not potentiate Treg development.

Since STAT3 transcriptional activity is critical for Th17 differentiation (50) we confirmed that our compound lacked any activity against the JAK/STAT3 pathway. We monitored the phosphorylation state of STAT3 in DMSO or Cpd A treated Ba/F3 cells. These cells stably express the human IL-21 receptor and are responsive to IL-21 stimulation by upregulating STAT3 phosphorylation. IL-21-induced STAT3 activity was monitored by flow cytometry. Inhibition of the JAK pathway by the pan-JAK inhibitor CP690550 blocked IL-21-induced STAT3 activity as evidenced by impaired phosphorylated (pY705)-STAT3 (Figure 3I). In contrast, Cpd A did not affect STAT3 phosphorylation compared to DMSO treatment (Figure 3I), suggesting that the JAK/STAT3 pathway is not directly targeted by Cpd A.

### Impaired Th17 Cell Signature Gene Expression After Inhibition of RORγt

**Figure 4:**
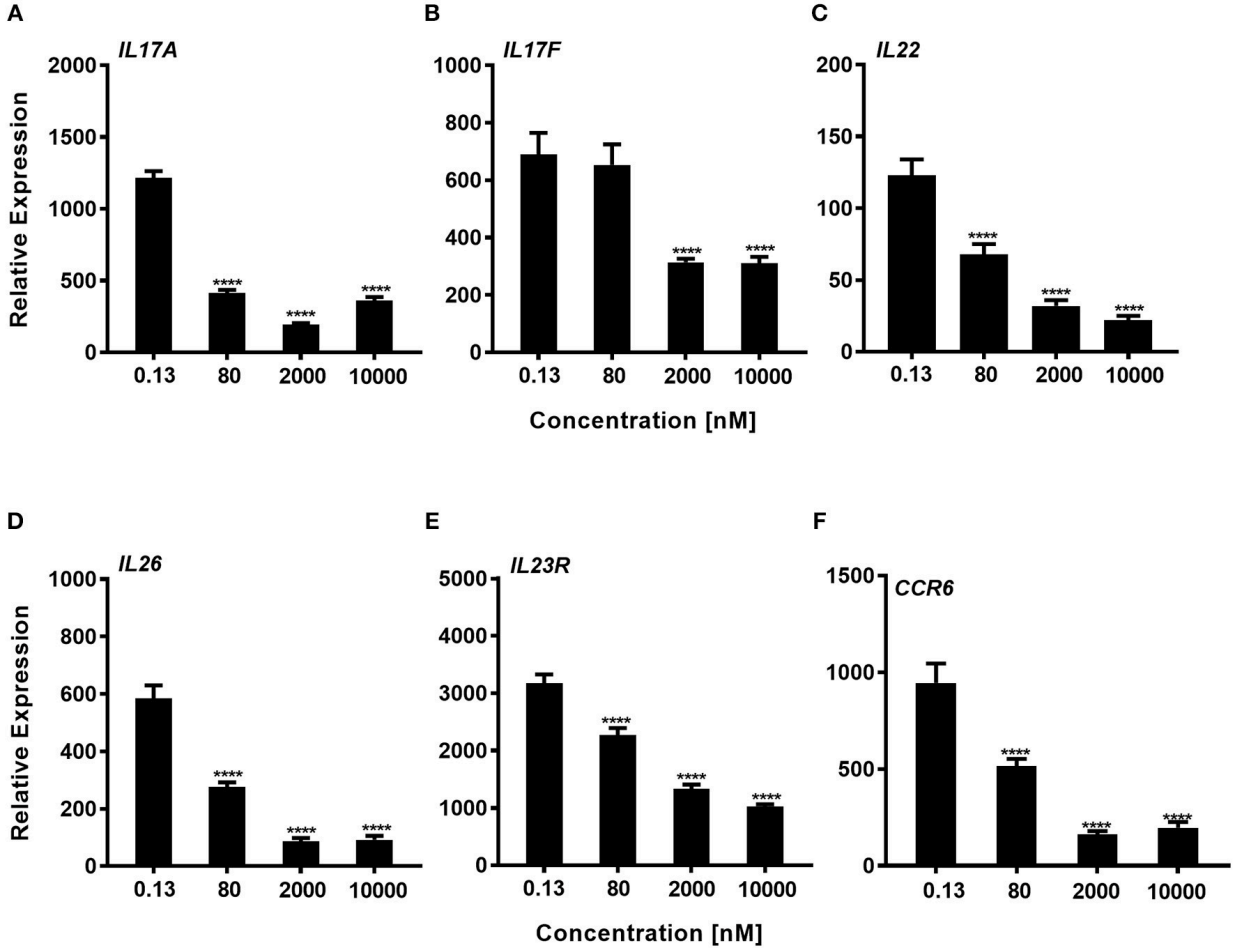
Impaired expression of RORγt-regulated genes in Th17 cells after Cpd A treatment. **(A–F)** Various Cpd A concentrations were added at the beginning of the Th17 cell polarizations and after 72 h, RORγt target gene expression was quantified by qRT-PCR. Gene expression was normalized to β-glucoronidase levels and expressed as arbitrary units. All graphs are representative from two experiments containing three technical replicates each. Error bars represent the SD. Significant inhibition was determined by one way ANOVA Dunnett's test, ****p < 0.0001.

We next explored whether expression of genes that are known to be regulated by RORγt were downregulated by the compound during the Th17 polarization. Using total CD4^+^ T-cells that were cultured for 72 h with a Th17 skewing cytokine mix we could show, that consistent with the effect of inhibition of IL-17A protein expression, Cpd A reduced RORγt signature gene expression in a concentration-dependent manner. In particular, pro-inflammatory Th17 pathway genes including *IL17A, IL17F, IL22, IL26, IL23R*, and *CCR6* were significantly attenuated by Cpd A (Figures 4A–F).

In summary, the RORγt low molecular weight compound reported here consistently and selectively suppressed not only IL-17A production by CD4^+^ T-cells, but also Th17-associated pro-inflammatory gene expression in human primary Th17 cells.

### RORγt Inhibition Leads to Attenuated Secondary Pro-inflammatory Mediator Responses in Keratinocytes and in Skin Immersion Cultures

**Figure 5:**
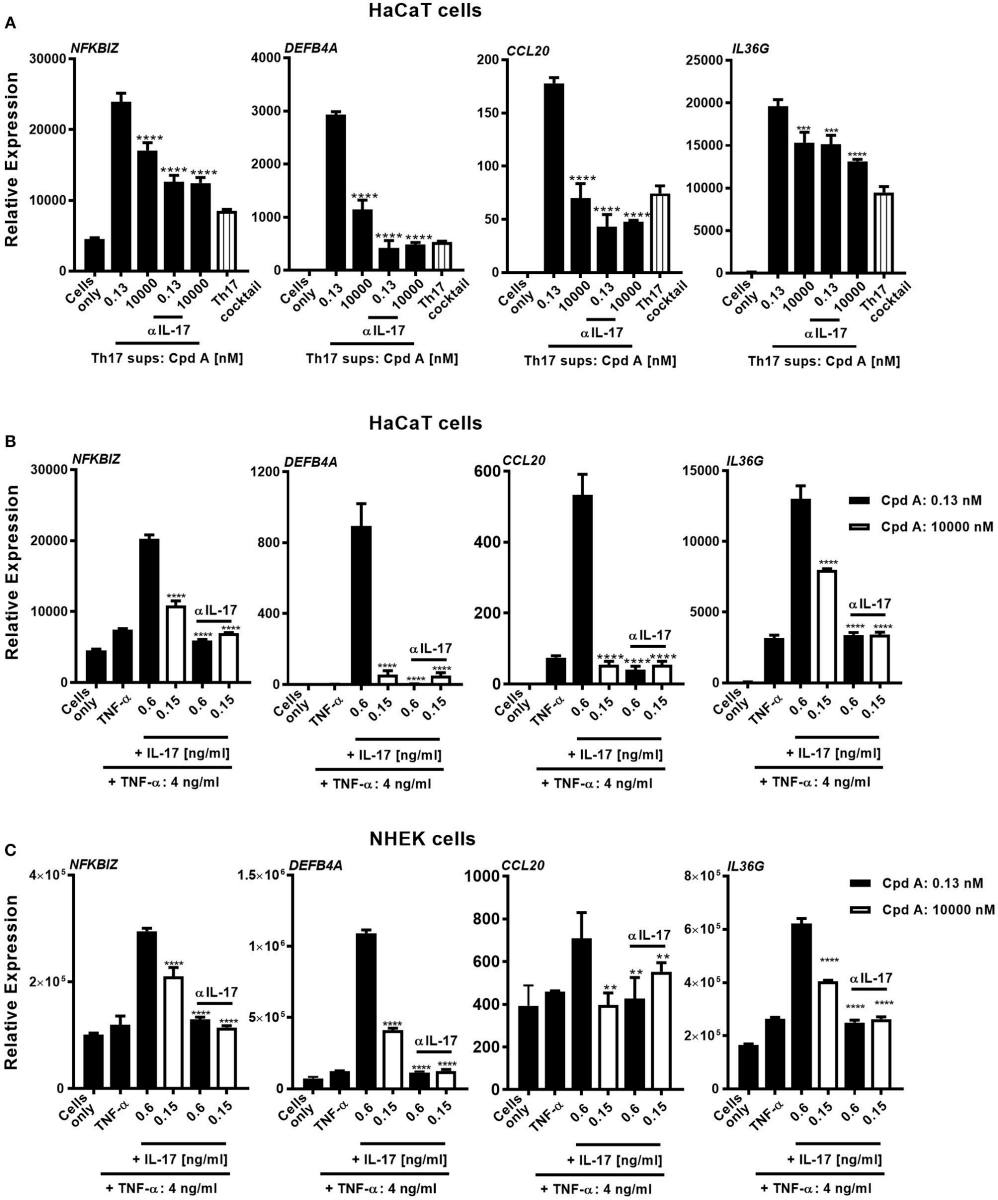
Cpd A attenuates IL-17-induced secondary responses in keratinocytes. **(A)** HaCaT keratinocytes were cultured for 24 h with supernatants derived from Cpd A treated Th17 cells either at a biologically inactive (0.13 nM) or at a pharmacologically active concentration (10 μM). To determine the effect of IL-17 in the conditioned supernatants on the HaCaT cell activation, half of the supernatants were pre-incubated with the IL-17 selective, neutralizing antibody Secukinumab (0.75 μg/ml). Alternatively, cells were left unstimulated or were exposed to Th17 cytokines that were used in the Th17 polarization assays. Keratinocytes were subjected to qRT-PCR analysis for determination of downstream IL-17-response genes, including *NFKBIZ, DEFB4A, CCL20*, and *IL36G*. **(B,C)** Defined IL-17A and TNFα concentrations which were measured in the Th17 cell supernatants originating from low-Cpd A-containing samples (IL-17: 0.6 ng/ml; TNFα 4 ng/ml) and from high-Cpd A-containing (IL-17: 0.15 ng/ml; TNFα: 4 ng/ml) or Secukinumab-treated samples were spiked into HaCaT cells **(B)** or alternatively into NHEK cell cultures originating from two donors **(C)**. To determine the impact of IL-17 in this cellular system, IL-17 was neutralized by addition of Secukinumab (0.75 μg/ml). Graphs are representative from three experiments containing triplicate readings. Significance between low-Cpd A-containing supernatants and between high-Cpd A and Secukinumab-containing samples was determined by ANOVA followed by Dunnett's test (***p < 0.005; ****p < 0.0001). Error bars represent the SD.

Human skin harbors a heterogeneous pool of immunocompetent cells, including DC subsets, Langerhans cells, CD4^+^, CD8^+^ T-cells, γδ T-cells, and innate lymphoid cells, that are able to produce pro-inflammatory cytokines/chemokines that can contribute to the pathogenesis of skin inflammatory disorders. Because of the good inhibitory profile of our compound we next investigated the impact of Th17 pathway inhibition on human keratinocyte responses triggered by Th17-derived cytokines present in supernatants originating from compound treated Th17 cells. For this, we selected cell supernatants originating from total CD4^+^ Th17 cells that were incubated with Cpd A for 72 h at a pharmacologically inactive concentration (0.13 nM) and at an active concentration (10 μM), containing 600 and 150 pg/ml of IL-17, respectively as depicted in Figure 3A. To determine the impact of IL-17 to induce keratinocyte responses, we depleted this cytokine from the supernatants using Secukinumab, a neutralizing IL-17-selective antibody prior to the co-culture experiment. IL-17 responsive transcripts that are upregulated in psoriasis were analyzed in keratinocytes and included *NFKBIZ, DEFB4, IL36G*, and *CCL20*. To evaluate the contribution of the polarizing Th17 cytokines present in the Th17-derived supernatants keratinocyte cultures were incubated with CD3/CD28-specific antibodies together with IL-6, IL-1β, TGF-β, and IL-23 at the same concentrations used in the Th17 polarization assays. Alternatively, recombinant human cytokines at the respective concentrations determined in the supernatants from Cpd A-treated cells were spiked into the HaCaT cultures to determine the contribution of other cytokines present in the Th17 supernatants on keratinocyte responses. A feature of IL-17 is that it acts in a synergistic way with other cytokines, particularly TNFα, to induce downstream gene/protein expression (51, 52). One abundant cytokine present in the Th17 supernatants was TNFα (4 ng/ml) whose level was not affected by RORγt inhibition. The Th17 supernatants contained similar IFN-γ concentrations and spike-in experiments revealed that this cytokine either alone or in combination with IL-17 and TNFα did not modulate expression of the IL-17 activated genes and therefore IFN-γ was omitted (data not shown). Upon 24 h activation with Th17-derived supernatants containing biologically inactive concentrations of Cpd A (0.13 nM), keratinocytes up-regulated expression of the IL-17-induced transcription factor *NFKBIZ*, the antimicrobial peptide *DEFB4A* and the chemokine *CCL20* and *IL36G* (Figure 5A). In contrast, supernatants obtained from Th17 samples that contained pharmacologically active concentrations of the RORγt inhibitor (10 μM) showed a marked reduction of these IL-17 signature genes. Consistent with the observation that the inactive and biologically active Cpd A-treated samples contained 150 and 600 pg/ml of IL-17, respectively, neutralization of IL-17 by Secukinumab led to further downregulation of these transcripts to the levels observed when the keratinocytes were incubated with the Th17 cytokines only (Figure 5A). The effect of Secukinumab to reduce these IL-17 activated genes was less pronounced in supernatants originating from cells treated with high Cpd A concentration. These results demonstrate that IL-17 was the main cytokine present in the Th17 supernatants that induced the expression of *NFKBIZ, DEFB4*, and *CCL20* in the keratinocyte cultures. Furthermore, the inhibitory capacity of Cpd A, when used at 10 μM, to attenuate IL-17 production and IL-17-induced gene activation was almost as pronounced as antibody-mediated neutralization of IL-17. *IL36G* expression was partially dependent on IL-17 because Secukinumab led to a moderate inhibition of *IL36G* indicating that other cytokines present in the Th17 supernatants or present in the Th17 cytokine mix induced *IL36G* gene expression. Similar results were obtained when recombinant IL-17A and TNFα cytokines were spiked into the keratinocyte cultures. *NFKBIZ, DEFB4, IL36G*, and *CCL20* expression was induced following stimulation of HaCaT cells with IL-17A/TNFα and the upregulation of these transcripts correlated directly with the IL-17A concentrations (600 pg/ml vs. 150 pg/ml) that were used for keratinocyte activation (Figure 5B). Co-stimulation of keratinocytes with 150 pg/ml of IL-17 which was the level determined in Th17 supernatants from cells treated with 10 μM of Cpd A induced only minimal *NFKBIZ, DEFB4, IL36G*, and *CCL20* expression. Pre-incubation of the cytokines with Secukinumab led to significantly impaired IL-17A/TNFα-mediated gene expression similar to the levels observed with TNFα-induced activation (Figure 5B). Because IL-17-mediated responses elicited by immortalized HaCaT cells may differ from those induced by primary keratinocytes, additional spike-in experiments using IL-17A/TNFα cytokines similar to those depicted in Figure 5B were performed using NHEKs. NHEKs responded in a similar way to IL-17A/TNFα stimulation by upregulating *NFKBIZ, DEFB4A, CCL20*, and *IL36G* gene expression (Figure 5C), although the IL-17A/TNFα stimulation index was lower in NHEKs mostly due to the higher basal gene expression levels in these cells. This was particular evident when *CCL20* transcript levels were analyzed. Upregulation of these transcripts correlated with the IL-17 concentrations that were used for the spike-in experiments (600 pg/ml vs. 150 pg/ml). Like in the HaCaT cells, neutralization of IL-17 by Secukinumab in NHEKs resulted in reduced gene expression similar to those observed induced after TNFα stimulation.

In summary, these results demonstrate that Th17 cell-derived IL17 is the key cytokine displaying pro-inflammatory activity in keratinocytes and that pharmacological inhibition of RORγt was efficacious to attenuate IL-17-driven keratinocyte responses.

**Figure 6:**
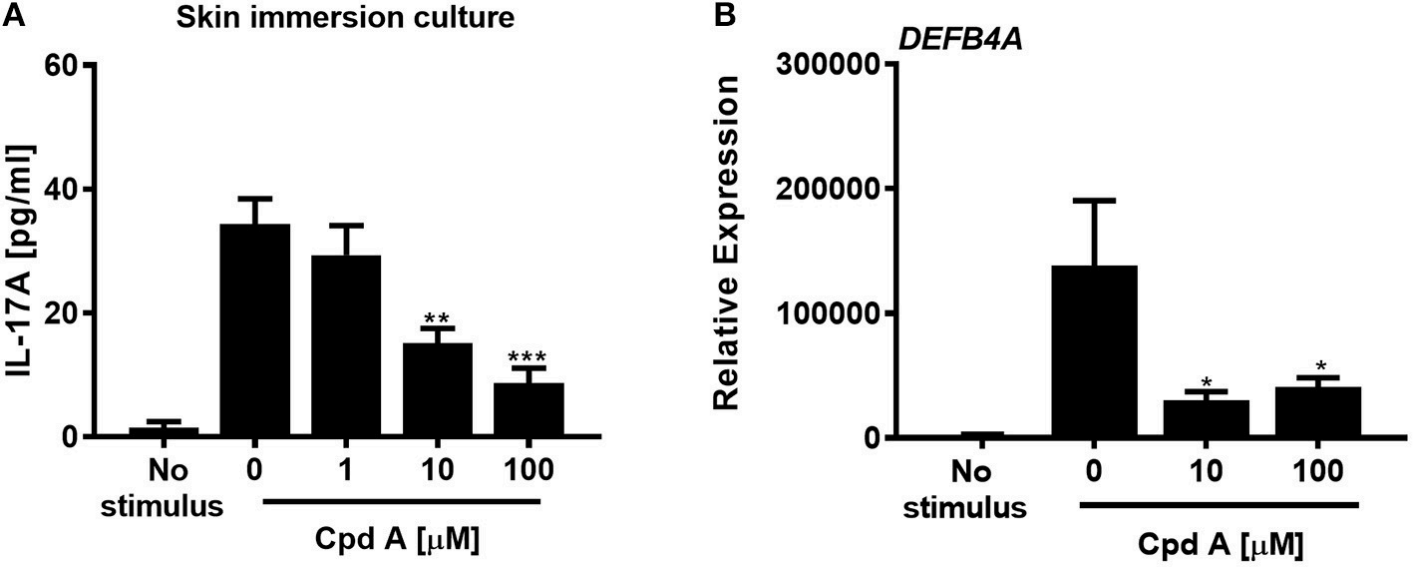
Cpd A inhibits IL-17A production by skin resident immunocompetent cells and impairs *DEFB4A* expression. **(A)** Full-thickness skin biopsies (8 mm diameter) were incubated with a Th17 cell skewing cytokine cocktail in the presence of various concentrations of Cpd A for 72 h followed by quantification of IL-17A production in the supernatants and qRT-PCR analysis of *DEFB4A* transcript expression in homogenized skin samples **(B)**. Graphs are representative from two independent experiments and each bar represents mean ± SEM from six replicates. Significant inhibition between no Cpd A treated vs. Cpd A treated samples was determined by ANOVA followed by Dunnett's test (*p < 0.05; **p < 0.01; ***p < 0.005). Error bars show the SEM.

In the next set of experiments we wanted to translate these findings and investigated whether addition of Cpd A was able to alter IL-17 pathway responses in freshly prepared full-thickness skin cultures. To recapitulate the predominant Th17/IL-23 cytokine signature occurring in psoriasis, immunocompetent skin-resident cells were activated with anti-CD3/CD28 antibodies together with a Th17 skewing cytokine cocktail, analogous to our blood-derived T-cell polarization studies. The full-thickness skin punch biopsies were incubated in the presence of Cpd A and IL-17 cytokine production and IL-17-induced *DEFB4A* expression in the skin was analyzed. Stimulation of skin-resident immune cells under Th17-polarizing conditions elicited significant IL-17A cytokine secretion in *ex vivo* human skin tissue, whereas skin samples that were left unstimulated barely produced IL-17A (Figure 6A). Addition of the RORγt inhibitor at the beginning of the activation period resulted in a concentration-dependent inhibition of IL-17A secretion by skin-resident immunocompetent cells (Figure 6A). In additional experiments, we investigated the impact of pharmacological inhibition of RORγt on secondary responses that may have been altered in the skin. Skin punches were homogenized and subjected to gene expression analysis by PCR. Correlating with the IL-17A inhibition pattern, h-BD2 gene expression was significantly down-regulated in Cpd A-treated skin punches (Figure 6B). Abundancy of *NFKBIZ, CCL20*, and *IL36G* transcripts were very low and results were inconclusive (data not shown).

These results suggest that selective RORγt inhibition during Th17 skewing conditions leads to attenuated IL-17 cytokine expression by immunocompetent skin-resident cells resulting in impaired secondary gene activation responses in keratinocytes and in *ex vivo* skin immersion cultures.

## Discussion

The nuclear hormone receptor RORγt plays a pivotal role in promoting the pro-inflammatory Th17/IL-17 pathway that has emerged as a major participant in inflammation and autoimmunity (1–3). Consequently, development of low molecular weight inhibitors against RORγt represents a highly promising therapeutic strategy to modulate diseases associated with the IL-17 immune axis. In this article, we describe a detailed *in vitro* characterization of an imidazopyridine RORγt inhibitor, Cpd A, that we recently identified from an extensive chemical optimization campaign (34). In a biochemical TR-FRET assay, the compound impaired the interaction between the RIP140 co-activator peptide and the RORγt-LBD and the inhibitory activity of the compound was mirrored in a cellular RORγt-LBD/GAL4 transactivation assay. These results indicate that Cpd A targets the LBD of RORγ resulting in disrupted co-activator recruitment and in impaired transcriptional activation. The compound not only showed potent inhibition when the RORγt-LBD was expressed, but showed similar potency toward full-length RORγt and inhibited RORE-driven gene reporter activity and endogenous IL-17 production by T-cells that were transduced with RORγt. In addition, the compound acted selectively toward the Th17 axis by blocking IL-17A production and did not interfere with RORγt unrelated pathways, such as IL-2 cytokine secretion. Moreover, the inhibitor showed good selectivity against RORγt and did not interfere with the activity of the closely related ROR family related transcription factors RORα or RORβ.

Mechanistically the compound could inhibit gene expression by preventing RORγt to bind to its consensus DNA binding motifs, the RORE sites located at target gene promoters, or by interfering with co-factor or transcription factor assembly at the RORγt-LBD while leaving the DNA binding activity of RORγt intact. We explored whether Cpd A affected engagement to the ROREs in oligonucleotide pull-down studies and we found that the inhibitor had no impact on the ability of the nuclear receptor to bind to the RORE elements. These results indicate that the mechanism of how Cpd A suppresses the transcriptional activity of RORγt likely involves blockade of co-factor recruitment to the LBD and/or compound-induced inhibition of transcription factor assembly necessary for gene transcription. A similar mode of action involving the LBD was also reported in studies of other low molecular weight inhibitors that did not interfere with the DNA-binding ability to RORγt target gene sequences (45, 53). We attempted to identify proteins whose interaction with RORγt may have been affected by Cpd A. Among these candidates were transcription/co-factors including transcriptional coactivator with PDZ motif (54), steroid receptor co-activators (55), p300, the transcriptional co-factor possessing intrinsic acetyltransferase activity (56, 57), hypoxia inducible factor 1α (56), or Runt-related transcription factor (58) that have been reported to interact with RORγt in various cellular systems. However, binding of these proteins to RORγt or to the RORE oligonucleotides remained intact following treatment of cells with Cpd A (data not shown). Further studies are necessary to identify the relevant co-activators/repressors or transcription factors that are affected by the RORγt inhibitor.

In line with an essential role of RORγt in Th17 polarization, Cpd A potently inhibited IL-17A production by human total CD4^+^ T-cells as well as naive and memory CD4^+^ T-cells while it did not interfere with the polarization of Th0, Th1, or Th2 cells. In addition, Cpd A impaired transcription of Th17-specific effector mediator genes, including *IL17F, IL22, IL26, IL23R*, and *CCR6* in differentiated human Th17 cells. Based on the inhibitory profile of Cpd A we speculate that RORγt inhibition may be more efficacious compared to existing modalities which target single cytokines or cytokine receptors implicated in the Th17 pathway. Thus, it would be interesting to compare the efficacy of Cpd A to other biologics, such as anti-IL-17, IL-23, or IL-22 antibody in *in vivo* inflammation models that have a dominant Th17-driven pathogenesis.

Targeting RORγt by a low molecular weight inhibitor may not only attenuate Th17 responses, but could also increase the frequencies of Tregs. Th17 and Treg cells are reciprocally interconnected and under IL-6, IL-21, and TGF-β1 prevalent cytokine conditions, RORγt expression is upregulated and Th17 cell development is favored. By contrast, in the presence of TGF-β1 and IL-2, FoxP3 expression is upregulated and the balance shifts toward Treg development (47, 59–62). Therefore, skewing the Th17/Treg cell ratio toward the Treg pathway as a result of RORγt inhibition could constitute an attractive therapeutic strategy resulting in additional benefit by inducing immune tolerance. We set up *in vitro* cultures to determine whether Cpd A affects FoxP3 expression under Treg inducing culture conditions by adding TGF-β1 and IL-2. Pharmacological inhibition of RORγt by Cpd A did not modify FoxP3 expression levels in CD4^+^CD25 high T-cells indicating that Treg development is neither favored nor compromised by our inhibitor. It has been reported that the expression levels of RORγt vs. FoxP3 are critical for the Th17/Treg balance (48). Overexpression of RORγt decreased FoxP3 expression, whereas knockdown of RORγt potentiated Foxp3 expression promoting Th17 and Treg development, respectively. We have preliminary evidence that Cpd A did not modify RORγt expression in our cultures (data not shown) and we speculate that sustained RORγt expression during Th17 differentiation may prevent a switch from Th17 to Treg development.

RORγt inhibition leads to reduced Th17 signature cytokine expression, including IL-22, IL-26, and IL-23 receptor that utilize the JAK/STAT3 pathway. As a consequence of the reduced Th17 signature cytokine levels, it is possible that STAT3 activity may also be inhibited during the Th17 differentiation process. Thus, in future experiments it would be interesting to monitor STAT3 phosphorylation in Cpd A treated cells during the Th17 culture period. We can exclude direct inhibition of STAT3 activity by Cpd A because IL-21-induced STAT3 phosphorylation in Ba/F3 cells was not affected by our RORγt inhibitor. It is possible that Cpd A acts on CD8^+^Tc17, ILC3 and γδ T-cells that express RORγt and contribute to tissue inflammation (1, 25). In a previous communication, it was shown by our group that a close analog belonging to the same series like Cpd A was equipotent to inhibit IL-17A production by primary human Tc17 as well as by γδ T-cells (44). Based on these results we speculate that it is likely that Cpd A would also exert activity toward these RORγt expressing cells. We were unable to confirm whether Cpd A attenuates IL-17A or IL-22 production by ILC3s because sufficient numbers of ILC3s from blood or from normal skin samples could not be isolated.

There is compelling evidence that the IL-17/IL-23 immune axis plays a pivotal role in the pathogenesis of psoriasis by promoting release of pro-inflammatory mediators by fibroblasts and keratinocytes, as well as inducing keratinocyte proliferation and inhibition of differentiation (10, 63, 64). Therapeutic targeting of IL-17 and IL-23 by antibody approaches has proven highly effective in the treatment of psoriasis (7, 16, 65, 66). To determine whether RORγt inhibition may impact downstream IL-17-driven keratinocyte responses we exposed HaCaT cells to supernatants derived from Cpd A treated Th17 cells, either at biologically active or inactive concentrations. We were able to show attenuated IL-17-associated signaling in keratinocyte cultures after incubation with Th17 cell-derived supernatants containing high Cpd A concentration. The Th17 cell supernatants contained various other cytokines that may have activated the keratinocytes. However, we could identify IL-17 as the main cytokine responsible for inducing *NFKBIZ, DEFB4*, and *CCL20* expression because IL-17 neutralization by Secukinumab reduced the transcripts to the same levels as observed with non-stimulated or Th17 cytokine cocktail-exposed keratinocytes. We utilized the HaCaT cell line and primary keratinocytes for spike-in experiments using recombinant IL-17A/TNFα cytokine concentrations measured at biologically inactive vs. active concentrations of Cpd A. Similar responses could be elicited by the cytokines in these cellular systems, although NHEKs were less responsive to stimulation compared to HaCaT cells. These results are in agreement with reports demonstrating that IL-17 and TNFα-induced responses, such as upregulation of *NFKBIZ, DEFB4* are comparable (67, 68), while it was shown that other responses, including expression pattern of cornified envelope-associated proteins differ between NHEKs and HaCaT cells (68). Samples containing pharmacologically active concentrations of the RORγt inhibitor (10 μM) showed a marked and significant reduction of these IL-17 signature genes compared to cells that were incubated with the cytokines at pharmacologically inactive concentrations. Secukinumab led to further downregulation of these transcripts.

Selective IL-17 blockade confirmed that IL-17A is the dominant driver to activate keratinocytes in our system. Studies are ongoing to evaluate the impact of other IL-17 isoforms, like IL-17AF, to induce pro-inflammatory responses in keratinocytes and whether IL-17AF expression is controlled by RORγt. In addition to keratinocytes, we were able to stimulate skin-resident immunocompetent cells *in situ* to produce IL-17A under culture conditions that mimicked the prevalent Th17/IL-23 cytokine profile in psoriasis. Consistent with the role of RORγt to induce IL-17A expression, addition of Cpd A inhibited IL-17A production by skin-resident cells and led to reduced *DEFB4A* expression in the skin samples.

Apart from Th17 cells, increased proportions of RORγt expressing NCR^+^ILC3 cells were present in lesional skin and in blood of psoriasis patients compared to healthy individuals (69, 70). These cells were cellular sources of IL-17 as well as IL-22, a cytokine that is regarded as a key driver of epidermal thickening. These results imply that NCR^+^ILC3s may contribute to psoriasis pathology. Future studies are required to identify and characterize the skin resident cell subsets that are targeted by our compound and to compare responses occurring in healthy vs. psoriatic skin tissue.

It has been reported that RORγ and RORα are expressed in all major compartments of human skin (71, 72). Based on molecular modeling and docking studies, their LBDs are predicted to interact with several vitamin D3 and lumisterol metabolites that are formed in the epidermis. While most of the active forms of vitamin D interact with the nuclear vitamin D receptor (VDR) (73), the noncalcemic secosteroids 1,25-dihydroxyvitamin D_3_, 20-hydroxy-and 20,23-dihydroxyvitamin D_3_, and several hydroxylumisterols were shown to be ligands for RORγ and RORα. When these vitamin D derivatives were added *ex vivo* to various cell cultures they acted as inverse agonists for RORγ and RORα as reflected by their ability to inhibit RORE-mediated transactivation as well as interfering with LXXLL-coactivator peptide binding to the RORγ/RORα LBDs in a mammalian 2-hybrid system (71, 72, 74). These results suggest that RORγ/RORα are involved in gene regulation in human skin. Emerging literature supports the notion that patients with psoriasis have lower serum vitamin D levels than control subjects (75–77) and there is evidence that the higher prevalence of vitamin D deficiency in chronic plaque psoriasis patients is inversely related to their PASI scores (78). It may be that under pathological conditions where availability of these endogenous RORγ inverse agonists are reduced, pharmacological inhibition by RORγ modulators may be a viable treatment option. However, further studies are required to determine the functional consequences of inhibition of skin-expressed RORγ, particularly in the context of psoriasis or other skin pathologies.

In summary, we identified a highly potent selective RORγt inhibitor that blocked RORγt-controlled Th17 signature genes and suppressed IL-17 cytokine production. Furthermore, attenuated IL-17 secretion limited expression of secondary keratinocyte-derived mediators that belong to the psoriasis signature. From a translational aspect, these results support the concept that RORγt inhibition achieved by the oral or topical route has considerable potential for the treatment of Th17-dependent diseases, particularly psoriasis and psoriatic arthritis.

## Data Availability

This manuscript contains previously unpublished data. The name of the repository and accession number are not available.

## Author Contributions

CG developed the study concept and wrote the manuscript along with input from SH and KK. CG, KK, SH, and DO designed experiments and interpreted the results and contributed to data collection and curation. JW and FE performed all the *in vitro* assays and were involved in data collection and analysis. All authors revised the manuscript and approved the final version.

### Conflict of Interest Statement

All authors work for Novartis. CG, KK, DO, and SH own shares and/or options from Novartis.

## References

[1] Korn T Bettelli E Oukka M Kuchroo VK. IL-17 and Th17 Cells. Annu Rev Immunol. (2009) 27:485–517. 10.1146/annurev.immunol.021908.132710 19132915

[2] McGeachy MJ Cua DJ. Th17 cell differentiation: the long and winding road. Immunity. (2008) 28:445–53. 10.1016/j.immuni.2008.03.001 18400187

[3] Bettelli E Korn T Oukka M Kuchroo VK. Induction and effector functions of T(H)17 cells. Nature. (2008) 453:1051–7. 10.1038/nature07036 18563156 PMC6280661

[4] Miossec P Kolls JK. Targeting IL-17 and TH17 cells in chronic inflammation. Nat Rev Drug Discov. (2012) 11:763–76. 10.1038/nrd3794 23023676

[5] Lock C Hermans G Pedotti R Brendolan A Schadt E Garren H. Gene-microarray analysis of multiple sclerosis lesions yields new targets validated in autoimmune encephalomyelitis. Nat Med. (2002) 8:500–8. 10.1038/nm0502-500 11984595

[6] Australo-Anglo-American Spondyloarthritis C Reveille JD Sims AM Danoy P Evans DM Leo P Genome-wide association study of ankylosing spondylitis identifies non-MHC susceptibility loci. Nat Genet. (2010) 42:123–7. 10.1038/ng.513 20062062 PMC3224997

[7] Patel DD Kuchroo VK. Th17 cell pathway in human immunity: lessons from genetics and therapeutic interventions. Immunity. (2015) 43:1040–51. 10.1016/j.immuni.2015.12.003 26682981

[8] Woo YR Cho DH Park HJ. Molecular mechanisms and management of a cutaneous inflammatory disorder: psoriasis. Int J Mol Sci. (2017) 18:E2684. 10.3390/ijms18122684 29232931 PMC5751286

[9] Fragoulis GE Siebert S McInnes IB. Therapeutic targeting of IL-17 and IL-23 cytokines in immune-mediated diseases. Annu Rev Med. (2016) 67:337–53. 10.1146/annurev-med-051914-021944 26565676

[10] Chiricozzi A Guttman-Yassky E Suarez-Farinas M Nograles KE Tian S Cardinale I. Integrative responses to IL-17 and TNF-alpha in human keratinocytes account for key inflammatory pathogenic circuits in psoriasis. J Invest Dermatol. (2011) 131:677–87. 10.1038/jid.2010.340 21085185

[11] Zhang L Yang XQ Cheng J Hui RS Gao TW. Increased Th17 cells are accompanied by FoxP3(+) Treg cell accumulation and correlated with psoriasis disease severity. Clin Immunol. (2010) 135:108–17. 10.1016/j.clim.2009.11.008 20006553

[12] Johansen C Usher PA Kjellerup RB Lundsgaard D Iversen L Kragballe K. Characterization of the interleukin-17 isoforms and receptors in lesional psoriatic skin. Br J Dermatol. (2009) 160:319–24. 10.1111/j.1365-2133.2008.08902.x 19016708

[13] Lowes MA Kikuchi T Fuentes-Duculan J Cardinale I Zaba LC Haider AS. Psoriasis vulgaris lesions contain discrete populations of Th1 and Th17 T cells. J Invest Dermatol. (2008) 128:1207–11. 10.1038/sj.jid.5701213 18200064

[14] Tonel G Conrad C Laggner U Di Meglio P Grys K McClanahan TK. Cutting edge: a critical functional role for IL-23 in psoriasis. J Immunol. (2010) 185:5688–91. 10.4049/jimmunol.1001538 20956338 PMC3776381

[15] Baeten D Baraliakos X Braun J Sieper J Emery PD van der Heijde D. Anti-interleukin-17A monoclonal antibody secukinumab in treatment of ankylosing spondylitis: a randomised, double-blind, placebo-controlled trial. Lancet. (2013) 382:1705–13. 10.1016/S0140-6736(13)61134-4 24035250

[16] Hueber W Patel DD Dryja T Wright AM Koroleva I Bruin G. Effects of AIN457, a fully human antibody to interleukin-17A, on psoriasis, rheumatoid arthritis, and uveitis. Sci Transl Med. (2010) 2:52ra72. 10.1126/scitranslmed.3001107 20926833

[17] Attia A Abushouk AI Ahmed H Gadelkarim M Elgebaly A Hassan Z. Safety and efficacy of brodalumab for moderate-to-severe plaque psoriasis: a systematic review and meta-analysis. Clin Drug Investig. (2017) 37:439–51. 10.1007/s40261-017-0500-9 28197901

[18] Ivanov II McKenzie BS Zhou L Tadokoro CE Lepelley A Lafaille JJ. The orphan nuclear receptor RORgammat directs the differentiation program of proinflammatory IL-17+ T helper cells. Cell. (2006) 126:1121–33. 10.1016/j.cell.2006.07.035 16990136

[19] Weaver CT Harrington LE Mangan PR Gavrieli M Murphy KM. Th17: an effector CD4 T cell lineage with regulatory T cell ties. Immunity. (2006) 24:677–88. 10.1016/j.immuni.2006.06.002 16782025

[20] Cook DN Kang HS Jetten AM. Retinoic acid-Related Orphan Receptors (RORs): regulatory functions in immunity, development, circadian rhythm, and metabolism. Nucl Receptor Res. (2015) 2:101185. 10.11131/2015/101185 26878025 PMC4750502

[21] Yang XO Pappu BP Nurieva R Akimzhanov A Kang HS Chung Y. T helper 17 lineage differentiation is programmed by orphan nuclear receptors ROR alpha and ROR gamma. Immunity. (2008) 28:29–39. 10.1016/j.immuni.2007.11.016 18164222 PMC2587175

[22] Hirota K Yoshitomi H Hashimoto M Maeda S Teradaira S Sugimoto N. Preferential recruitment of CCR6-expressing Th17 cells to inflamed joints via CCL20 in rheumatoid arthritis and its animal model. J Exp Med. (2007) 204:2803–12. 10.1084/jem.20071397 18025126 PMC2118525

[23] Ciofani M Madar A Galan C Sellars M Mace K Pauli F. A validated regulatory network for Th17 cell specification. Cell. (2012) 151:289–303. 10.1016/j.cell.2012.09.016 23021777 PMC3503487

[24] Cua DJ Tato CM. Innate IL-17-producing cells: the sentinels of the immune system. Nat Rev Immunol. (2010) 10:479–89. 10.1038/nri2800 20559326

[25] Gaffen SL Jain R Garg AV Cua DJ. The IL-23-IL-17 immune axis: from mechanisms to therapeutic testing. Nat Rev Immunol. (2014) 14:585–600. 10.1038/nri3707 25145755 PMC4281037

[26] Yen HR Harris TJ Wada S Grosso JF Getnet D Goldberg MV. Tc17 CD8 T cells: functional plasticity and subset diversity. J Immunol. (2009) 183:7161–8. 10.4049/jimmunol.0900368 19917680 PMC3082359

[27] Sutton CE Mielke LA Mills KH. IL-17-producing gammadelta T cells and innate lymphoid cells. Eur J Immunol. (2012) 42:2221–31. 10.1002/eji.201242569 22949320

[28] Spits H Cupedo T. Innate lymphoid cells: emerging insights in development, lineage relationships, and function. Annu Rev Immunol. (2012) 30:647–75. 10.1146/annurev-immunol-020711-075053 22224763

[29] Leppkes M Becker C Ivanov II Hirth S Wirtz S Neufert C. RORgamma-expressing Th17 cells induce murine chronic intestinal inflammation via redundant effects of IL-17A and IL-17F. Gastroenterology. (2009) 136:257–67. 10.1053/j.gastro.2008.10.018 18992745

[30] Pantelyushin S Haak S Ingold B Kulig P Heppner FL Navarini AA. Rorgammat+ innate lymphocytes and gammadelta T cells initiate psoriasiform plaque formation in mice. J Clin Invest. (2012) 122:2252–6. 10.1172/JCI61862 22546855 PMC3366412

[31] Cyr P Bronner SM Crawford JJ. Recent progress on nuclear receptor RORgamma modulators. Bioorg Med Chem Lett. (2016) 26:4387–93. 10.1016/j.bmcl.2016.08.012 27542308

[32] Pandya VB Kumar S Sachchidanand Sharma R Desai RC Combating autoimmune diseases with retinoic acid receptor-Related Orphan Receptor-gamma (RORgamma or RORc) inhibitors: hits and misses. J Med Chem. (2018) 61:10976–95. 10.1021/acs.jmedchem.8b00588 30010338

[33] Zhang Y Luo XY Wu DH Xu Y. ROR nuclear receptors: structures, related diseases, and drug discovery. Acta Pharmacol Sin. (2015) 36:71–87. 10.1038/aps.2014.120 25500868 PMC4571318

[34] Hintermann S Guntermann C Mattes H Carcache DA Wagner J Vulpetti A. Synthesis and biological evaluation of new triazolo- and imidazolopyridine RORgammat inverse agonists. ChemMedChem. (2016) 11:2640–8. 10.1002/cmdc.201600500 27902884

[35] Kallen J Izaac A Be C Arista L Orain D Kaupmann K. Structural states of RORgammat: X-ray elucidation of molecular mechanisms and binding interactions for natural and synthetic compounds. ChemMedChem. (2017) 12:1014–21. 10.1002/cmdc.201700278 28590087

[36] Kolbinger F Loesche C Valentin MA Jiang X Cheng Y Jarvis P. beta-Defensin 2 is a responsive biomarker of IL-17A-driven skin pathology in patients with psoriasis. J Allergy Clin Immunol. (2017) 139:923–32 e928. 10.1016/j.jaci.2016.06.038 27502297

[37] Gallais Serezal I Classon C Cheuk S Barrientos-Somarribas M Wadman E Martini E. Resident T cells in resolved psoriasis steer tissue responses that stratify clinical outcome. J Invest Dermatol. (2018) 138:1754–63. 10.1016/j.jid.2018.02.030 29510191

[38] Keermann M Koks S Reimann E Prans E Abram K Kingo K. Transcriptional landscape of psoriasis identifies the involvement of IL36 and IL36RN. BMC Genomics. (2015) 16:322. 10.1186/s12864-015-1508-2 25897967 PMC4405864

[39] D'Erme AM Wilsmann-Theis D Wagenpfeil J Holzel M Ferring-Schmitt S Sternberg S. IL-36gamma (IL-1F9) is a biomarker for psoriasis skin lesions. J Invest Dermatol. (2015) 135:1025–32. 10.1038/jid.2014.532 25525775

[40] Bertelsen T Iversen L Johansen C. The human IL-17A/F heterodimer regulates psoriasis-associated genes through IkappaBzeta. Exp Dermatol. (2018) 27:1048–52. 10.1111/exd.13722 29938836

[41] Wu NL Huang DY Tsou HN Lin YC Lin WW. Syk mediates IL-17-induced CCL20 expression by targeting Act1-dependent K63-linked ubiquitination of TRAF6. J Invest Dermatol. (2015) 135:490–8. 10.1038/jid.2014.383 25202827

[42] Kumar N Lyda B Chang MR Lauer JL Solt LA Burris TP. Identification of SR2211: a potent synthetic RORgamma-selective modulator. ACS Chem Biol. (2012) 7:672–7. 10.1021/cb200496y 22292739 PMC3331898

[43] Flanagan ME Blumenkopf TA Brissette WH Brown MF Casavant JM Shang-Poa C. Discovery of CP-690,550:a potent and selective Janus kinase (JAK) inhibitor for the treatment of autoimmune diseases and organ transplant rejection. J Med Chem. (2010) 53:8468–84. 10.1021/jm1004286 21105711

[44] Guntermann C Piaia A Hamel ML Theil D Rubic-Schneider TA Del Rio-Espinola A. Retinoic-acid-orphan-receptor-C inhibition suppresses Th17 cells and induces thymic aberrations. JCI Insight. (2017) 2:e91127. 10.1172/jci.insight.91127 28289717 PMC5333964

[45] Guendisch U Weiss J Ecoeur F Riker JC Kaupmann K Kallen J. Pharmacological inhibition of RORgammat suppresses the Th17 pathway and alleviates arthritis *in vivo*. PLoS ONE. (2017) 12:e0188391. 10.1371/journal.pone.0188391 29155882 PMC5695821

[46] Collingwood TN Urnov FD Wolffe AP. Nuclear receptors: coactivators, corepressors and chromatin remodeling in the control of transcription. J Mol Endocrinol. (1999) 23:255–75. 10.1677/jme.0.0230255 10601972

[47] Kleinewietfeld M Hafler DA. The plasticity of human Treg and Th17 cells and its role in autoimmunity. Semin Immunol. (2013) 25:305–12. 10.1016/j.smim.2013.10.009 24211039 PMC3905679

[48] Burgler S Mantel PY Bassin C Ouaked N Akdis CA Schmidt-Weber CB. RORC2 is involved in T cell polarization through interaction with the FOXP3 promoter. J Immunol. (2010) 184:6161–9. 10.4049/jimmunol.0903243 20427770

[49] Bennett CL Christie J Ramsdell F Brunkow ME Ferguson PJ Whitesell L. The immune dysregulation, polyendocrinopathy, enteropathy, X-linked syndrome (IPEX) is caused by mutations of FOXP3. Nat Genet. (2001) 27:20–1. 10.1038/83713 11137993

[50] Hirahara K Ghoreschi K Laurence A Yang XP Kanno Y O'Shea JJ. Signal transduction pathways and transcriptional regulation in Th17 cell differentiation. Cytokine Growth Factor Rev. (2010) 21:425–34. 10.1016/j.cytogfr.2010.10.006 21084214 PMC3182452

[51] Amatya N Garg AV Gaffen SL. IL-17 signaling: the Yin and the Yang. Trends Immunol. (2017) 38:310–22. 10.1016/j.it.2017.01.006 28254169 PMC5411326

[52] Hartupee J Liu C Novotny M Li X Hamilton T. IL-17 enhances chemokine gene expression through mRNA stabilization. J Immunol. (2007) 179:4135–41. 10.4049/jimmunol.179.6.4135 17785852

[53] Xiao S Yosef N Yang J Wang Y Zhou L Zhu C. Small-molecule RORgammat antagonists inhibit T helper 17 cell transcriptional network by divergent mechanisms. Immunity. (2014) 40:477–89. 10.1016/j.immuni.2014.04.004 24745332 PMC4066874

[54] Geng J Yu S Zhao H Sun X Li X Wang P The transcriptional coactivator TAZ regulates reciprocal differentiation of TH17 cells and Treg cells. Nat Immunol. (2017) 18:800–12. 10.1038/ni.3748 28504697

[55] Xie H Sadim MS Sun Z. RORgammat recruits steroid receptor coactivators to ensure thymocyte survival. J Immunol. (2005) 175:3800–9. 10.4049/jimmunol.175.6.3800 16148126

[56] Dang EV Barbi J Yang HY Jinasena D Yu H Zheng Y. Control of T(H)17/T(reg) balance by hypoxia-inducible factor 1. Cell. (2011) 146:772–84. 10.1016/j.cell.2011.07.033 21871655 PMC3387678

[57] Wu Q Nie J Gao Y Xu P Sun Q Yang J. Reciprocal regulation of RORgammat acetylation and function by p300 and HDAC1. Sci Rep. (2015) 5:16355. 10.1038/srep16355 26549310 PMC4817527

[58] Zhang F Meng G Strober W. Interactions among the transcription factors Runx1, RORgammat and Foxp3 regulate the differentiation of interleukin 17-producing T cells. Nat Immunol. (2008) 9:1297–306. 10.1038/ni.1663 18849990 PMC4778724

[59] Bettelli E Carrier Y Gao W Korn T Strom TB Oukka M. Reciprocal developmental pathways for the generation of pathogenic effector TH17 and regulatory T cells. Nature. (2006) 441:235–8. 10.1038/nature04753 16648838

[60] Ivanov II Zhou L Littman DR. Transcriptional regulation of Th17 cell differentiation. Semin Immunol. (2007) 19:409–17. 10.1016/j.smim.2007.10.011 18053739 PMC2696342

[61] Ichiyama K Yoshida H Wakabayashi Y Chinen T Saeki K Nakaya M. Foxp3 inhibits RORgammat-mediated IL-17A mRNA transcription through direct interaction with RORgammat. J Biol Chem. (2008) 283:17003–8. 10.1074/jbc.M801286200 18434325

[62] Zhou L Lopes JE Chong MM Ivanov II Min R Victora GD. TGF-beta-induced Foxp3 inhibits T(H)17 cell differentiation by antagonizing RORgammat function. Nature. (2008) 453:236–40. 10.1038/nature06878 18368049 PMC2597437

[63] Witte E Kokolakis G Witte K Philipp S Doecke WD Babel N. IL-19 is a component of the pathogenetic IL-23/IL-17 cascade in psoriasis. J Invest Dermatol. (2014) 134:2757–67. 10.1038/jid.2014.308 25046339

[64] Volpe E Servant N Zollinger R Bogiatzi SI Hupe P Barillot E. A critical function for transforming growth factor-beta, interleukin 23 and proinflammatory cytokines in driving and modulating human T(H)-17 responses. Nat Immunol. (2008) 9:650–7. 10.1038/ni.1613 18454150

[65] Gordon KB Strober B Lebwohl M Augustin M Blauvelt A Poulin Y. Efficacy and safety of risankizumab in moderate-to-severe plaque psoriasis (UltIMMa-1 and UltIMMa-2): results from two double-blind, randomised, placebo-controlled and ustekinumab-controlled phase 3 trials. Lancet. (2018) 392:650–61. 10.1016/S0140-6736(18)31713-6 30097359

[66] Leonardi C Matheson R Zachariae C Cameron G Li L Edson-Heredia E. Anti-interleukin-17 monoclonal antibody ixekizumab in chronic plaque psoriasis. N Engl J Med. (2012) 366:1190–9. 10.1056/NEJMoa1109997 22455413

[67] Muller A Hennig A Lorscheid S Grondona P Schulze-Osthoff K Hailfinger S. IkappaBzeta is a key transcriptional regulator of IL-36-driven psoriasis-related gene expression in keratinocytes. Proc Natl Acad Sci USA. (2018) 115:10088–93. 10.1073/pnas.1801377115 30224457 PMC6176600

[68] Seo MD Kang TJ Lee CH Lee AY Noh M. HaCaT keratinocytes and primary epidermal keratinocytes have different transcriptional profiles of cornified envelope-associated genes to T helper cell cytokines. Biomol Ther. (2012) 20:171–6. 10.4062/biomolther.2012.20.2.171 24116291 PMC3792214

[69] Teunissen MBM Munneke JM Bernink JH Spuls PI Res PCM Te Velde A. Composition of innate lymphoid cell subsets in the human skin: enrichment of NCR(+) ILC3 in lesional skin and blood of psoriasis patients. J Invest Dermatol. (2014) 134:2351–60. 10.1038/jid.2014.146 24658504

[70] Villanova F Flutter B Tosi I Grys K Sreeneebus H Perera GK. Characterization of innate lymphoid cells in human skin and blood demonstrates increase of NKp44+ ILC3 in psoriasis. J Invest Dermatol. (2014) 134:984–91. 10.1038/jid.2013.477 24352038 PMC3961476

[71] Slominski AT Kim TK Takeda Y Janjetovic Z Brozyna AA Skobowiat C. RORalpha and ROR gamma are expressed in human skin and serve as receptors for endogenously produced noncalcemic 20-hydroxy- and 20,23-dihydroxyvitamin D. FASEB J. (2014) 28:2775–89. 10.1096/fj.13-242040 24668754 PMC4062828

[72] Slominski AT Kim TK Hobrath JV Janjetovic Z Oak ASW Postlethwaite A. Characterization of a new pathway that activates lumisterol *in vivo* to biologically active hydroxylumisterols. Sci Rep. (2017) 7:11434. 10.1038/s41598-017-10202-7 28900196 PMC5595834

[73] Bikle DD. Vitamin D and the skin: physiology and pathophysiology. Rev Endocr Metab Disord. (2012) 13:3–19. 10.1007/s11154-011-9194-0 21845365 PMC3687803

[74] Slominski AT Kim TK Hobrath JV Oak ASW Tang EKY Tieu EW. Endogenously produced nonclassical vitamin D hydroxy-metabolites act as “biased” agonists on VDR and inverse agonists on RORalpha and RORgamma. J Steroid Biochem Mol Biol. (2017) 173:42–56. 10.1016/j.jsbmb.2016.09.024 27693422 PMC5373926

[75] Orgaz-Molina J Magro-Checa C Arrabal-Polo MA Raya-Alvarez E Naranjo R Buendia-Eisman A. Association of 25-hydroxyvitamin D with metabolic syndrome in patients with psoriasis: a case-control study. Acta Derm Venereol. (2014) 94:142–5. 23995104 10.2340/00015555-1642

[76] Soleymani T Hung T Soung J. The role of vitamin D in psoriasis: a review. Int J Dermatol. (2015) 54:383–92. 10.1111/ijd.12790 25601579

[77] Staberg B Oxholm A Klemp P Christiansen C. Abnormal vitamin D metabolism in patients with psoriasis. Acta Derm Venereol. (1987) 67:65–8. 2436417

[78] Ricceri F Pescitelli L Tripo L Prignano F. Deficiency of serum concentration of 25-hydroxyvitamin D correlates with severity of disease in chronic plaque psoriasis. J Am Acad Dermatol. (2013) 68:511–12. 10.1016/j.jaad.2012.10.051 23394917

