# Supplementary material for: Antagonizing Retinoic Acid-Related-Orphan Receptor Gamma Activity Blocks the T Helper 17/Interleukin-17 Pathway Leading to Attenuated Pro-inflammatory Human Keratinocyte and Skin Responses
Source: Front Immunol. 2019 Mar 26;10:577. doi: 10.3389/fimmu.2019.00577 (PMC6443933; doi:10.3389/fimmu.2019.00577)
Supplement: Supplementary file 1 [file Table_1.DOCX]

Antagonizing Retinoic acid-related-orphan receptor gamma Activity Blocks the T Helper 17/Interleukin-17 Pathway Leading to Attenuated Pro-inflammatory Human Keratinocyte and Skin Responses

Florence Ecoeur^†1^, Jessica Weiss^†1^, Klemens Kaupmann^1^, Samuel Hintermann^2^, David Orain^2^, Christine Guntermann^1*^

^1^Autoimmunity, Transplantation, and Inflammation Disease Area, Novartis Institutes for BioMedical Research, Basel, Switzerland

^2^Global Discovery Chemistry, Novartis Institutes for BioMedical Research, Basel, Switzerland

^*^Correspondence: Christine Guntermann, [christine.guntermann@novartis.com](mailto:christine.guntermann@novartis.com)

Supplementary Material

**Supplementary Figure 1.** Synthesis of Cpd A: the starting material (designated compound **12** in *Hintermann et al* (1) was synthesized as described in the Material and Methods.

**REFERENCES**

1. Hintermann, S., C. Guntermann, H. Mattes, D. A. Carcache, J. Wagner, A. Vulpetti, A. Billich, J. Dawson, K. Kaupmann, J. Kallen, R. Stringer, and D. Orain. 2016. Synthesis and Biological Evaluation of New Triazolo- and Imidazolopyridine RORgammat Inverse Agonists. *ChemMedChem* 11: 2640-2648.
